# Effectiveness of the Ad26.COV2.S vaccine in health-care workers in South Africa (the Sisonke study): results from a single-arm, open-label, phase 3B, implementation study

**DOI:** 10.1016/S0140-6736(22)00007-1

**Published:** 2022-03-19

**Authors:** Linda-Gail Bekker, Nigel Garrett, Ameena Goga, Lara Fairall, Tarylee Reddy, Nonhlanhla Yende-Zuma, Reshma Kassanjee, Shirley Collie, Ian Sanne, Andrew Boulle, Ishen Seocharan, Imke Engelbrecht, Mary-Ann Davies, Jared Champion, Tommy Chen, Sarah Bennett, Selaelo Mametja, Mabatlo Semenya, Harry Moultrie, Tulio de Oliveira, Richard John Lessells, Cheryl Cohen, Waasila Jassat, Michelle Groome, Anne Von Gottberg, Engelbert Le Roux, Kentse Khuto, Dan Barouch, Hassan Mahomed, Milani Wolmarans, Petro Rousseau, Debbie Bradshaw, Michelle Mulder, Jessica Opie, Vernon Louw, Barry Jacobson, Pradeep Rowji, Jonny G Peter, Azwi Takalani, Jackline Odhiambo, Fatima Mayat, Simbarashe Takuva, Lawrence Corey, Glenda E Gray, Sisonke Protocol Team, Sisonke Study Team, William Brumskine, Nivashnee Naicker, Disebo Makhaza, Vimla Naicker, Logashvari Naidoo, Elizabeth Spooner, Elane van Nieuwenhuizen, Kathryn Mngadi, Maphoshane Nchabeleng, James Craig Innes, Katherine Gill, Friedrich Georg Petrick, Shaun Barnabas, Sharlaa Badal-Faesen, Sheetal Kassim, Scott Hayden Mahoney, Erica Lazarus, Anusha Nana, Rebone Molobane Maboa, Philip Kotze, Johan Lombaard, Daniel Rudolf Malan, Sheena Kotze, Phuthi Mohlala, Amy Ward, Graeme Meintjes, Dorothea Urbach, Faeezah Patel, Andreas Diacon, Khatija Ahmed, Coert Grobbelaar, Pamela Mda, Thozama Dubula, Angelique Luabeya, Musawenkosi Bhekithemba Mamba, Lesley Burgess, Rodney Dawson

**Affiliations:** a The Desmond Tutu HIV Centre, Cape Town, South Africa; b Division of Allergy and Clinical Immunology, Cape Town, South Africa; c Centre of Infectious Disease Epidemiology and Research, School of Public Health and Family Medicine, Cape Town, South Africa; d Department of Pathology, Cape Town, South Africa; e Division of Clinical Haematology, Department of Medicine, Cape Town, South Africa; f Faculty of Health Sciences, and Allergy and Immunology Unit, Cape Town, South Africa; g University of Cape Town, Cape Town, South Africa; h Doris Duke Medical Research Institute, University of KwaZulu-Natal, Durban, South Africa; i Nelson R Mandela School of Medicine, Centre for the AIDS Programme of Research in South Africa, University of KwaZulu-Natal, Durban, South Africa; j KwaZulu-Natal Research Innovation, School of Laboratory Medicine and Medical Sciences, University of KwaZulu-Natal, Durban, South Africa; k HIV Prevention Research Unit, Cape Town, South Africa; l South Africa Medical Research Council, Cape Town, South Africa; m Department of Paediatrics and Child Health, University of Pretoria, Pretoria, South Africa; n School of Health Systems and Public Health, Faculty of Health Sciences, University of Pretoria, Pretoria, South Africa; o King's Global Health Institute, King's College London, London, UK; p Biostatistics Research Unit, South African Medical Research Council, Durban, South Africa; q Discovery Health, Sandton, South Africa; r Clinical HIV Research Unit, Faculty of Health Sciences, University of the Witwatersrand, Witwatersrand, South Africa; s School of Public Health, Faculty of Health Sciences, University of the Witwatersrand, Witwatersrand, South Africa; t School of Pathology, Faculty of Health Sciences, University of the Witwatersrand, Witwatersrand, South Africa; u Right to Care, Houghton South Africa; v Western Cape Government Health, Cape Town, South Africa; w Wellcome Centre for Infectious Diseases Research in Africa, Cape Town, South Africa; x Right to Care, Centurion, South Africa; y Western Cape Government: Health Centre for Infectious Disease Epidemiology and Research, Cape Town, South Africa; z Medscheme, Cape Town, South Africa; aa Government Employees Medical Scheme, Pretoria, South Africa; ab Centre for Tuberculosis, National Institute for Communicable Diseases of the National Health Laboratory Service, Johannesburg, South Africa; ac Centre for Respiratory Diseases and Meningitis, National Institute for Communicable Diseases of the National Health Laboratory Service, Johannesburg, South Africa; ad National Institute for Communicable Diseases, Sandringham, South Africa; ae Division of Public Health Surveillance and Response, National Institute for Communicable Diseases, Johannesburg, South Africa; af Hutchinson Center Research Institute of South Africa (HCRISA), Chris Hani Baragwanath Academic Hospital, Soweto, South Africa; ag Center for Virology and Vaccine Research, Beth Israel Deaconess Medical Center, Harvard Medical School, Boston, MA, USA; ah Metro Health Services, Western Cape Government Health, Cape Town, South Africa; ai Division of Health Systems and Public Health, Department of Global Health, Faculty of Medicine and Health Sciences, Stellenbosch University, Cape Town, South Africa; aj National Department of Health, Pretoria, South Africa; ak South African Medical Research Council, Tygerberg, South Africa; al National Health Laboratory Service, Cape Town, South Africa; am Groote Schuur Hospital, Cape Town, South Africa; an Neurology Association of South Africa, The Southern African Society of Thrombosis and Haemostasis, Johannesburg, South Africa; ao Perinatal HIV Research Unit (PHRU), Chris Hani Baragwanath Academic Hospital, University of the Witwatersrand, Witwatersrand, South Africa; ap Vaccine and Infectious Disease Division, Fred Hutchinson Cancer Research Center, Seattle, WA, USA

## Abstract

**Background:**

We aimed to assess the effectiveness of a single dose of the Ad26.COV2.S vaccine (Johnson & Johnson) in health-care workers in South Africa during two waves of the South African COVID-19 epidemic.

**Methods:**

In the single-arm, open-label, phase 3B implementation Sisonke study, health-care workers aged 18 years and older were invited for vaccination at one of 122 vaccination sites nationally. Participants received a single dose of 5 × 10^10^ viral particles of the Ad26.COV2.S vaccine. Vaccinated participants were linked with their person-level data from one of two national medical insurance schemes (scheme A and scheme B) and matched for COVID-19 risk with an unvaccinated member of the general population. The primary outcome was vaccine effectiveness against severe COVID-19, defined as COVID-19-related admission to hospital, hospitalisation requiring critical or intensive care, or death, in health-care workers compared with the general population, ascertained 28 days or more after vaccination or matching, up to data cutoff. This study is registered with the South African National Clinical Trial Registry, DOH-27-022021-6844, ClinicalTrials.gov, NCT04838795, and the Pan African Clinical Trials Registry, PACTR202102855526180, and is closed to accrual.

**Findings:**

Between Feb 17 and May 17, 2021, 477 102 health-care workers were enrolled and vaccinated, of whom 357 401 (74·9%) were female and 119 701 (25·1%) were male, with a median age of 42·0 years (33·0–51·0). 215 813 vaccinated individuals were matched with 215 813 unvaccinated individuals. As of data cutoff (July 17, 2021), vaccine effectiveness derived from the total matched cohort was 83% (95% CI 75–89) to prevent COVID-19-related deaths, 75% (69–82) to prevent COVID-19-related hospital admissions requiring critical or intensive care, and 67% (62–71) to prevent COVID-19-related hospitalisations. The vaccine effectiveness for all three outcomes were consistent across scheme A and scheme B. The vaccine effectiveness was maintained in older health-care workers and those with comorbidities including HIV infection. During the course of the study, the beta (B.1.351) and then the delta (B.1.617.2) SARS-CoV-2 variants of concerns were dominant, and vaccine effectiveness remained consistent (for scheme A plus B vaccine effectiveness against COVID-19-related hospital admission during beta wave was 62% [95% CI 42–76] and during delta wave was 67% [62–71], and vaccine effectiveness against COVID-19-related death during beta wave was 86% [57–100] and during delta wave was 82% [74–89]).

**Interpretation:**

The single-dose Ad26.COV2.S vaccine shows effectiveness against severe COVID-19 disease and COVID-19-related death after vaccination, and against both beta and delta variants, providing real-world evidence for its use globally.

**Funding:**

National Treasury of South Africa, the National Department of Health, Solidarity Response Fund NPC, The Michael & Susan Dell Foundation, The Elma Vaccines and Immunization Foundation, and the Bill & Melinda Gates Foundation.

## Introduction

Since March, 2020, South Africa has experienced four distinct waves of the COVID-19 pandemic, each characterised by different circulating SARS-CoV-2 variants of concern.^1^ South Africa has the eighth highest number of excess deaths due to COVID-19 by population globally, and has high levels of previous SARS-CoV-2 infection.^2^, ^3^ Vaccine supply has been low and health-care workers have been severely affected due to their proximity to patients.^4^, ^5^ On the basis of the efficacy results of the ENSEMBLE trial,^6^ which was conducted when the SARS-CoV-2 beta (B.1.351) variant was circulating in South Africa, the single-dose Johnson & Johnson Ad26.COV2.S COVID-19 vaccine was made available to health-care workers, as part of the Sisonke study.^6^, ^7^ The Sisonke study started before emergency use authorisation and the national roll-out of this vaccine in South Africa.^7^

**Research in context**

**Evidence before this study**

To date, there has been a paucity of real-world effectiveness data for the Ad26.COV2.S vaccine (Johnson & Johnson), especially in settings where the beta (B.1.351) or delta (B.1.617.2) SARS-CoV-2 variants of concern are circulating. A number of smaller studies have confirmed the vaccine effectiveness of the Ad26.COV2.S vaccine in the USA. These studies have been mostly in settings where the delta variant is not the dominant variant. Health-care workers are important essential workers who are also highly exposed to SARS-CoV-2. There is an urgent need to expand the Ad26.COV2.S vaccine evidence base in the face of the delta variant, given the reports of reduced effectiveness of other COVID-19 vaccines in settings where variants such as beta or delta are predominantly circulating. Additionally, more data on vaccine effectiveness in people living with HIV is needed, but also in these epidemiological settings.

**Added value of this study**

The Sisonke study, which was conducted among health-care workers in South Africa, is the largest and only study to date to assess effectiveness overall, and against the beta and delta variants of SARS-CoV-2, of the Ad26.COV2.S vaccine in health-care workers. Our analysis suggests that vaccine effectiveness determined when the beta variant of concern was dominant in the country was maintained against the delta variant, showing high rates of protection against COVID-19-related death and hospitalisation. Because our study population comprised a large number of health-care workers with HIV, we also provide additional evidence that the Ad26.COV2.S vaccine provided protection in this sub-population.

**Implications of all the available evidence**

Our study, together with other real-world effectiveness studies, affirm the effectiveness of the Ad26.COV2.S vaccine against severe COVID-19, as defined by COVID-19-related hospitalisation and death, even in the presence of the delta variant. Our ability to demonstrate protection in people living with HIV is a critical finding for HIV burdened regions of the world. This study provides additional support for the rapid deployment of this vaccine globally.

The primary objective of the Sisonke study was to assess the effectiveness of the single-dose Ad26.COV2.S vaccine to prevent COVID-19-related admission to hospital (hereafter referred to as hospitalisation), hospitalisation requiring critical care unit (CCU) or intensive care unit (ICU) admission, and death in health-care workers. Additionally, with a large proportion of the population living with HIV in South Africa, in post-hoc analyses, we were able to assess vaccine effectiveness among health-care workers living with HIV. Here, we report an analysis of the Sisonke study, in which we assessed vaccine effectiveness in this vaccinated health-care worker population compared with a cohort of unvaccinated individuals from the general population, matched for COVID-19 risk factors.

## Methods

### Study design and population

In this analysis of the Sisonke study, we used a matched cohort design, similar to that described by Dagan and colleagues.^8^ In the Sisonke single-arm, open-label, phase 3B, implementation study, health-care workers across all regions of South Africa aged 18 years and older were invited, via the national online electronic vaccination data system (EVDS), to register for vaccination and were then directed to give electronic informed consent to participate in the study before receiving the vaccination at one of 122 national vaccination sites. Each vaccination site was linked to a Sisonke clinical research team approved by the South African Health Products Regulatory Authority (SAHPRA) and an affiliated human research ethics committee.

The definition of health-care worker was broad, but patient-facing and front-line workers were prioritised for participation up to May 11, 2021, after which inclusion criteria were expanded to include non-patient-facing health-care workers, support staff, and administrative staff, as well as community health workers, staff in care homes, and funeral workers.^7^ Full eligibility criteria have been reported elsewhere.^7^ Briefly, pregnant women were not approved by SAHPRA to participate during the period of enrolment and for health-care workers with a history of severe adverse reaction associated with a vaccine or severe allergic reaction (eg, anaphylaxis) to any component of the vaccine, eligibility was determined after consultation with a protocol safety review team. Following a pause called by the US Food and Drug Administration on April 13, 2021, to review unusual clotting events in vaccine recipients in the USA, participants with a history of major venous or arterial thrombosis with thrombocytopenia and those with a history of heparin-induced thrombocytopenia were no longer recruited. Thereafter, participants with a chronic history of severe clotting disorders were only included after approval by the protocol safety review team. Participants were not specifically tested for SARS-CoV-2 antibody status before vaccination.

The protocol for the Sisonke study was reviewed and approved by the SAHPRA, and all health research ethics committees associated with Sisonke clinical research sites. Approvals and data sharing agreements enabled the research team to access anonymised unvaccinated datasets after matching. A protocol safety team met each week to review all safety events, breakthrough SARS-CoV-2 infections, and all-cause deaths. The Protocol Safety Committee provided independent oversight of the study. Electronic informed consent was obtained from all health-care workers, and consent was not needed from matched unvaccinated individuals.

### Procedures

After clinical assessment for COVID-19 symptoms by a vaccinator, a single dose of 5 × 10^10^ viral particles of Ad26.COV2.S vaccine (Johnson & Johnson) was administered into the non-dominant deltoid muscle of each participating health-care worker. Follow-up was through both active and passive surveillance, with prospective follow-up being for 2 years. Vaccinees were provided with details of an online self-administered data collection tool, toll-free telephone number, or email address through which they could report any adverse events or breakthrough SARS-CoV-2 infections. Vaccinees were sent three SMS message notifications after vaccination reminding them to report any adverse events. These notifications were sent on the day of vaccination and on days 7 and 14. Additionally, any breakthrough SARS-CoV-2 infections, COVID-19-related hospitalisations, and deaths were linked through the COVID-19 Notifiable Medical Conditions Sentinel Surveillance List, the DATCOV database that contains data on individuals admitted to hospital with COVID-19, and the National Population Register (appendix pp 4–5). All events were followed up with SMS messages, telephone interviews with vaccinees, family members, or health providers, and review of medical records. Safety and tolerability have been previously reported^9^ and are not included in this report.

To facilitate a detailed assessment of vaccine effectiveness, we used data from two large national medical scheme administrators or managed-care organisations (health-care schemes administered by Discovery Health, hereafter referred to as scheme A, and Government Employees Medical Scheme, MedScheme, hereafter referred to as scheme B), in which person-level data were available for vaccinees and for members of the general population. We used the EVDS to provide information on vaccination status of all people vaccinated in South Africa, including those who contributed unvaccinated at-risk time to analyses, allowing right censoring. On each successive day of the Sisonke vaccination roll-out, each newly vaccinated health-care worker for whom person-level data were available through linkage with the two schemes (A and B) was matched to a member of the general population. For scheme B, matched individuals were restricted to essential workers who might carry a high risk of contracting COVID-19. For a sensitivity analysis, we used person-level data from the Western Cape provincial health department database of health-care worker employees to match the province's cohort of vaccinated health-care workers with unvaccinated health-care workers. Where data sharing agreements were completed, datasets were completed using deterministic linkage based on the South African civil identification number or passport number for foreign nationals. Deterministic linkage was completed in secure environments and data were anonymised.

Four matching variables were standardised across the two scheme datasets: age, sex, geographical location using district, and total number of risk factors for severe COVID-19 aligned with definitions from the US Centres for Disease Control and Prevention (CDC; eg, diabetes, hypertension, HIV, cardiovascular disease, chronic liver disease, chronic renal disease, cancer, chronic respiratory disease, neurological disorders, overweight or obesity, severe mental disorders, and solid organ transplant recipient).^10^ Additionally, participants and controls were matched on socioeconomic status, according to medical plan option for scheme A and income level for scheme B, and individuals in scheme A were also matched by previous SARS-CoV-2 infection (could not be used for matching in scheme B due to a paucity of data). There were fewer variables available in the Western Cape provincial database, and so vaccinated and unvaccinated individuals were matched on: age, sex, occupational group, number of previous negative COVID-19 tests (a proxy for health-seeking behaviour), and previous SARS-CoV-2 infection. The success of matching vaccinated individuals to similar counterparts was assessed by comparing means of key characteristics not used for matching (acceptable if standardised mean difference was <0·1), and risk of our prespecified outcomes in the 6–13 days before full vaccine effectiveness is expected.

Clinical characteristics were derived from medical insurance data for schemes A and B and baseline and clinical characteristics for the full Sisonke study cohort were self-reported on the EVDS. Clinical characteristics were not available in the Western Cape provincial dataset. Breakthrough SARS-CoV-2 infection outcomes were monitored in the whole vaccinated health-care worker cohort. Breakthrough SARS-CoV-2 infections were adjudicated on the basis of information provided by participants through an online self-administered data collection tool or participant-led telephone interviews with participants, family members, or health providers. Medical records were retrieved to validate clinical data, to assess severe outcomes, and to investigate potential COVID-19 deaths that were identified through the national vital register. Breakthrough infections were defined as SARS-CoV-2 infections occurring 28 days or longer after vaccination and with a laboratory-confirmed PCR or antigen test. Disease severity was classified using an adaptation of the WHO Clinical Progression Scale, defined as follows: mild disease, which comprised individuals with asymptomatic SARS-CoV-2 infection and symptomatic disease managed at home with no oxygen therapy or with a hospital stay of less than 24 h; moderate disease, requiring hospital care for more than 24 h or oxygen therapy provided via nasal prongs or face mask; severe disease, requiring high flow oxygen therapy, non-invasive ventilation, mechanical ventilation or vasopressors, dialysis, or extracorporeal membrane oxygenation; and any breakthrough infection that resulted in death.

Additionally, when health-care workers reported breakthrough infections, nasopharyngeal samples were taken and, when viable, sent for viral genotyping at a central laboratory that forms part of the network of genomic surveillance of South Africa (NGSA). The NGSA database was used to determine the beta and delta dominant periods in South Africa.

### Outcomes

The primary objective of the study was to assess the effectiveness of Ad26.COV2.S vaccine on severe COVID-19, assessed via the primary outcomes of COVID-19-related hospitalisation, COVID-19-related hospitalisation requiring CCU or ICU admission, or COVID-19 related death 28 days or longer after vaccination, in health-care workers compared with the general unvaccinated population in South Africa. Full definitions of outcomes are in the appendix (p 6).

Secondary endpoints were: incidence of laboratory-confirmed SARS CoV-2 infection on PCR or antigen test as indicated by self-report, health insurance claims and records, and validation through linkage to national laboratory records; rates of severe disease in vaccinated health-care workers who were found to be RT-PCR positive after vaccination, as measured by hospitalisation, ICU admission, and death; genetic diversity of breakthrough infection virus, as determined by whole-genome sequencing; prevalence of SARS-CoV-2 seropositivity at baseline; levels of neutralising antibodies, non-neutralising antibodies, and T-cell immunity in the blood samples of health-care workers who had breakthrough infections; anti-SARS-CoV-2 neutralising antibody titres and T-cell responses among vaccinees in groups of interest; rates of asymptomatic infection at baseline and follow-up using SARS CoV-2 PCR and antibody testing in a subset of health-care workers; the proportion of health-care workers who registered for vaccination on the EVDS; and the rate of vaccination in health-care workers per week of the study. Analyses of these secondary endpoints are ongoing and will be presented elsewhere.^9^

### Statistical analysis

For each individual, the time at risk included in the analysis started on the date of vaccination or matching. Censoring of both people in a pair occurred if the study period ended (July 17, 2021), the unvaccinated counterpart was vaccinated, or either individual left the scheme or died due to a reason other than COVID-19. All newly vaccinated health-care workers were eligible for inclusion in the study, even if they had previously acted as a matched control at an earlier date, although newly vaccinated individuals for whom no matched counterpart could be identified were excluded from the analysis. A person could only act as an unvaccinated control to a vaccinee once.

For the Western Cape provincial dataset, scheme A, scheme B, and scheme A and B combined, we estimated vaccine effectiveness as the relative reduction in the incidence (number of outcome events divided by time at risk) for the vaccinated versus unvaccinated group from day 28 after vaccination or after matching, as appropriate, until the end of follow-up (maximum of 150 days). We visualised the data by cumulative incidence functions using a Kaplan-Meier approach.

We calculated uncertainties in vaccine effectiveness and cumulative incidence estimates using percentile bootstrap confidence intervals (500 bootstraps), to account for both sampling variability and variability from the stochastic matching process. We calculated point estimates and descriptive statistics (frequencies, proportions, number of events, person-years, and cumulative risks for the Kaplan-Meier curves) by averaging over bootstraps. We calculated 95% CIs by averaging over bootstraps. A bootstrap replication contributed to the estimation if each subcohort (Western Cape, scheme A, or scheme B) had at least some minimum number of events over the period of interest (defined as a minimum of ten events for schemes A and B and three events for the Western Cape provincial dataset). We did subgroup analyses of the primary outcome according to baseline characteristics, and by dominant variant of concern (post hoc). We used calendar period as a proxy for when each of the two variants of concern, beta and delta, were the dominant circulating variant. Using data from the NGSA, we determined that the beta variant was dominant in the region from the beginning of the study period until May 17, 2021, and thereafter the delta variant was expanding and had exceeded 25% of sequences (appendix pp 2–3).

We did several sensitivity analyses. We compared primary outcomes in vaccinated health-care workers who could be matched with unvaccinated health-care workers in the Western Cape provincial dataset. We did analyses for the periods of 0–5, 6–13, and 14–27 days using matched pairs in which both individuals were still at risk (ie, not infected) at the beginning of each period of interest to explore potential confounders in mismatching or due to differences in health-seeking behaviours. In a further sensitivity analysis, we constructed covariate balance loveplots to show the standardised difference in means between vaccinated and unvaccinated groups for the different CDC risk criteria for scheme A and B.

We did a descriptive analysis of breakthrough infections in vaccinated health-care workers, by age, sex, and admission status. We also did descriptive analyses of death in vaccinated health-care workers.

Analyses were done in SAS (version 9.4), Stata SE (version 17), and R (version 4.05). The trial was registered at South African National Clinical Trial Registry, DOH-27-022021-6844, ClinicalTrials.gov, NCT04838795, and the Pan African Clinical Trials Registry, PACTR202102855526180.

### Role of the funding source

The funders of the study had no role in the study design, data collection, data analysis, data interpretation or writing of the report.

## Results

**Figure 1:**
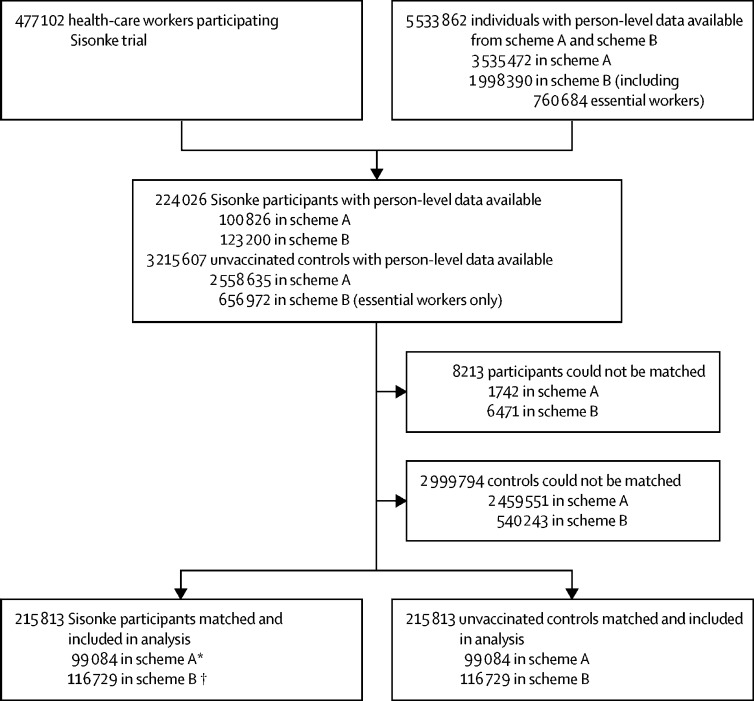
Trial profile Sisonke participants were matched with unvaccinated controls on age, sex, number of comorbidities, geographical location (health district), socioeconomic status (by scheme plan option for scheme A and income level for scheme B), and, for scheme A, previous SARS-CoV-2 infection (could not be matched for scheme B due to paucity of data available).*Includes 2027 individuals who were also included in the Western Cape provincial dataset. †Includes 13 135 individuals who were also included in the Western Cape provincial dataset.

Between Feb 17 and May 17, 2021, 477 102 health-care worker were enrolled and vaccinated. 357 401 (74·9%) health-care workers were female and 119 701 (25·1%) were male, with a median age of 42·0 years (33·0–51·0). Recruitment occurred between the second (Nov 15, 2020, to Feb 6, 2021) and third (May 9 to Sept 18, 2021) epidemiological waves of the COVID-19 epidemic in South Africa (appendix p 3). Vaccinated participants in the Sisonke trial with person-level data available in one of two insurance schemes (scheme A or scheme B) were matched with unvaccinated individuals with person-level data available in each of these schemes (figure 1). Similarly, Sisonke participants with data in the Western Cape provincial database were matched with unvaccinated health-care workers (appendix p 11). There was overlap between health-care workers represented in the provincial dataset and the scheme A and B datasets. The number of health-care workers who were included in the scheme-based subgroups and in the provincial dataset were 2027 in scheme A and 13 135 in scheme B.

Of the 224 026 vaccinees and 3 215 607 unvaccinated counterparts with person-level data in the two insurance schemes, 8213 (4%) vaccinees could not be matched, leaving 215 813 matched individuals available for analysis. The baseline demographic and clinical characteristics of all the Sisonke vaccine recipients and of unvaccinated individuals included in the analysis of each subcohort are shown in table 1. There are a higher number of health-care workers in Gauteng, KwaZulu Natal, Western and Eastern Cape than in other provinces, which was reflected in the numbers of health-care workers vaccinated in each province. Health-care workers in scheme A were younger than those in scheme B and the majority in scheme A were situated in Gauteng (appendix pp 12–15). The clinical characteristics of the vaccinees in the two schemes were similar except for HIV seropositivity, which was higher among those in scheme B, and overweight and obesity, which was higher among those in scheme A. The Western Cape cohort had a higher prevalence of documented previous SARS-CoV-2 infection than in schemes A and B, with nurses accounting for two-fifths of the cohort, and management and support staff another fifth (appendix p 16). There were no significant differences (ie, greater than 0·10) in CDC risk criteria observed between vaccinated and unvaccinated groups in schemes A and B (appendix p 17).

**Table 1:** Baseline demographic and clinical characteristics of all vaccinated health-care workers and by matched subcohort, and for the matched unvaccinated populations

		**All vaccinated health-care workers (N=477 102)**	**Matched vaccinated health-care workers**				**Matched unvaccinated individuals**			
			Scheme A and B (n=215 813)	Scheme A (n=99 084)	Scheme B (n=116 729)	Western Cape provincial dataset (n=19 951)	Scheme A and B (n=215 813)	Scheme A (n=99 084)	Scheme B (n=116 729)	Western Cape provicinal dataset (n=19 951)
Sex										
	Female	357 401 (74·9%)	165 452 (76·7%)	72 093 (72·8%)	93 359 (80·0%)	14 641 (73·4%)	165 452 (76·7%)	72 093 (72·8%)	93 359 (80·0%)	14 641 (73·4%)
	Male	119 701 (25·1%)	50 362 (23·3%)	26 992 (27·2%)	23 370 (20·0%)	5310 (26·6%)	50 362 (23·3%)	26 992 (27·2%)	23 370 (20·0%)	5310 (26·6%)
Follow-up time, days		109 (72·0–129·0)	108·6 (71·3–128·3)	108·7 (67·5–129·8)	108·4 (74·2–126·6)	72·1 (16·0–118·0)	108·3 (71·1–128·1)	108·1 (67·0–129·4)	108·4 (74·4–126·6)	72·0 (16·0–118·0)
Age, years										
	Median	42·0 (33·0–51·0)	42·9 (35·0–51·4)	39·7 (31·6–49·2)	44·9 (37·8–52·6)	41·0 (24·0–61·0)	42·4 (34·6–50·9)	39·2 (31·1–48·7)	44·5 (37·4–52·2)	41·0 (24·0–60·0)
	<18	0	0	0	0	0	48 (<0·1%)	48 (<0·1%)	0	0
	18–39	209 411 (43·9%)	81 336 (37·7%)	47 430 (47·9%)	33 906 (29·1%)	9089 (45·6%)	84 529 (39·2%)	48 884 (49·3%)	35 645 (30·5%)	9284 (46·5%)
	40–49	136 967 (28·7%)	67 746 (31·4%)	26 499 (26·7%)	41 247 (35·3%)	5489 (27·5%)	66 947 (31·0%)	26 046 (26·3%)	40 901 (35·0%)	5353 (26·8%)
	50–59	96 235 (20·2%)	48 978 (22·7%)	16 505 (16·7%)	32 473 (27·8%)	4593 (23·0%)	48 157 (22·3%)	16 073 (16·2%)	32 084 (27·5%)	4641 (23·3%)
	60–69	29 181 (6·1%)	15 678 (7·3%)	6988 (7·1%)	8690 (7·4%)	780 (3·9%)	14 222 (6·6%)	6512 (6·6%)	7710 (6·6%)	673 (3·4%)
	70–79	4698 (1·0%)	1875 (0·9%)	1492 (1·5%)	383 (0·3%)	0	1729 (0·8%)	1368 (1·4%)	361 (0·3%)	0
	≥80	610 (0·1%)	200 (0·1%)	170 (0·2%)	30 (<0·1%)	0	181 (0·1%)	153 (0·2%)	28 (<0·1%)	0
Geographical location										
	Eastern Cape	57 673 (12·1%)	20 163 (9·3%)	5671 (5·7%)	14 492 (12·4%)	..	19 482 (·09%)	5384 (5·4%)	14 098 (12·1%)	..
	Free State	24 182 (5·1%)	11 276 (5·2%)	3672 (3·7%)	7604 (6·5%)	..	11 597 (5·4%)	3733 (3·8%)	7864 (6·7%)	..
	Gauteng	124 865 (26·2%)	64 490 (29·9%)	42 136 (42·5%)	22 354 (19·2%)	..	66 465 (30·8%)	43 498 (43·9%)	22 967 (19·7%)	..
	Kwazulu-Natal	92 689 (19·4%)	41 553 (19·3%)	16 152 (16·3%)	25 401 (21·8%)	..	40 944 (19·0%)	15 939 (16·1%)	25 005 (21·4%)	..
	Limpopo	33 222 (7·0%)	16 051 (7·4%)	1955 (2·0%)	14 096 (12·1%)	..	16 028 (7·4%)	2115 (2·1%)	13 913 (11·9%)	..
	Mpumalanga	20 362 (4·3%)	9115 (4·2%)	2283 (2·3%)	6832 (5·9%)	..	9393 (4·4%)	2361 (2·4%)	7032 (6·0%)	..
	North West	23 046 (4·8%)	10 650 (4·9%)	2253 (2·3%)	8397 (7·2%)	..	11 225 (5·2%)	2407 (2·4%)	8818 (7·6%)	..
	Northern Cape	9343 (2·0%)	2999 (1·4%)	834 (0·8%)	2165 (1·9%)	..	3117 (1·4%)	863 (0·9%)	2254 (1·9%)	..
	Western Cape	91 720 (19·2%)	38 839 (18·0%)	23 451 (23·7%)	15 388 (13·2%)	19 951 (100%)	36 881 (17·1%)	22 103 (22·3%)	14 778 (12·7%)	19 951 (100%)
	Unallocated	0	678 (0·3%)	678 (0·7%)	0	0	681 (0·3%)	681 (0·7%)	0	0
Number of risk factors for severe COVID-19										
	0	..	141 692 (65·7%)	66 741 (67·4%)	74 951 (64·2%)	..	141 692 (65·7%)	66 741 (67·4%)	74 951 (64·2%)	..
	1	..	54 548 (25·3%)	24 495 (24·7%)	30 053 (25·8%)	..	54 548 (25·3%)	24 495 (24·7%)	30 053 (25·7%)	..
	2	..	16 041 (7·4%)	6200 (6·3%)	9841 (8·4%)	..	16 041 (7·4%)	6200 (6·3%)	9841 (8·4%)	..
	≥3	..	3533 (1·6%)	1649 (1·7%)	1884 (1·6%)	..	3533 (1·6%)	1649 (1·7%)	1884 (1·6%)	..
Risk factors for severe COVID-19										
	Diabetes	28 058 (5·9%)	13 012 (6·0%)	4207 (4·2%)	8805 (7·5%)		12 306 (5·7%)	4281 (4·3%)	8025 (6·9%)	
	Hypertension	74 370 (15·6%)	32 768 (15·2%)	12 421 (12·5%)	20 347 (17·4%)	..	33 266 (15·4%)	12 508 (12·6%)	20 758 (17·8%)	..
	HIV	39 383 (8·3%)	23 752 (11·0%)	4720 (4·8%)	19 032 (16·3%)	..	22 148 (10·3%)	3537 (3·6%)	18 611 (15·9%)	..
	Cardiovascular disease	3430 (0·7%)	2360 (1·1%)	1343 (1·4%)	1017 (0·9%)	..	2405 (1·1%)	1394 (1·4%)	1011 (0·9%)	..
	Chronic liver disease	..	282 (0·1%)	280 (0·3%)	2 (<0·1%)		275 (0·1%)	273 (0·3%)	2 (<0·1%)	
	Chronic renal disease	..	278 (0·1%)	154 (0·2%)	124 (0·1%)	..	380 (0·2%)	221 (0·2%)	159 (0·1%)	..
	Cancer	1364 (0·3%)	1673 (0·8%)	909 (0·9%)	764 (0·7%)	..	1882 (0·9%)	1017 (1·0%)	865 (0·7%)	..
	Chronic respiratory disease	1733 (0·4%)	7905 (3·7%)	5244 (5·3%)	2661 (2·3%)	..	7701 (3·6%)	4762 (4·8%)	2939 (2·5%)	..
	Neurological disorders	..	1463 (0·7%)	870 (0·9%)	593 (0·5%)	..	1986 (0·9%)	1197 (1·2%)	789 (0·7%)	..
	Overweight or obesity	..	11 058 (5·1%)	10 080 (10·2%)	978 (0·8%)		11 244 (5·2%)	10 312 (10·4%)	932 (0·8%)	
	Severe mental disorders	..	2700 (1·3%)	1958 (2·0%)	742 (0·6%)	..	3633 (1·7%)	2621 (2·6%)	1012 (0·9%)	..
	Solid organ transplant recipient	..	50 (<0·1%)	50 (0·1%)	0	..	50 (<0·1%)	50 (0·1%)	0	..
History of COVID-19*										
	Ever had a COVID-19 test	..	129 655 (60·1%)	88 691 (89·5%)	40 964 (35·1%)	11 886 (59·6%)	133 238 (61·7%)	88 691 (89·5%)	44 547 (38·2%)	11 886 (59·6%)
	Documented previous SARS-CoV-2 infection (wave 1)†	..	13 742 (6·4%)	5666 (5·7%)	8076 (6·9%)	2692 (13·5%)	12 792 (5·9%)	5616 (5·7%)	7176 (6·1%)	1446 (7·2%)
	Documented previous SARS-CoV-2 infection (wave 2)	..	9780 (4·5%)	4727 (4·8%)	5053 (4·3%)	2030 (10·2%)	10 837 (5%)	4777 (4·8%)	6060 (5·2%)	1169 (5·9%)

Data are n (%) or median (IQR).

* Before date of vaccination or matching.

† Infection before Oct 1, 2020.

As of data cutoff (July 17, 2021), among matched vaccinees, 302 COVID-19-related hospitalisations occurred (153 in scheme A, 149 in scheme B), 63 COVID-19-related hospital admissions requiring critical or intensive care occurred (19 in scheme A and 44 in scheme B), and 28 COVID-19-related deaths occurred. Among matched unvaccinated members of the general population**,** 897 COVID-19-related hospitalisations occurred (444 in scheme A, 453 in scheme B), 256 COVID-19-related hospital admissions requiring critical or intensive care occurred (110 in scheme A and 146 in scheme B), and 163 COVID-19 related deaths occurred.

**Figure 2:**
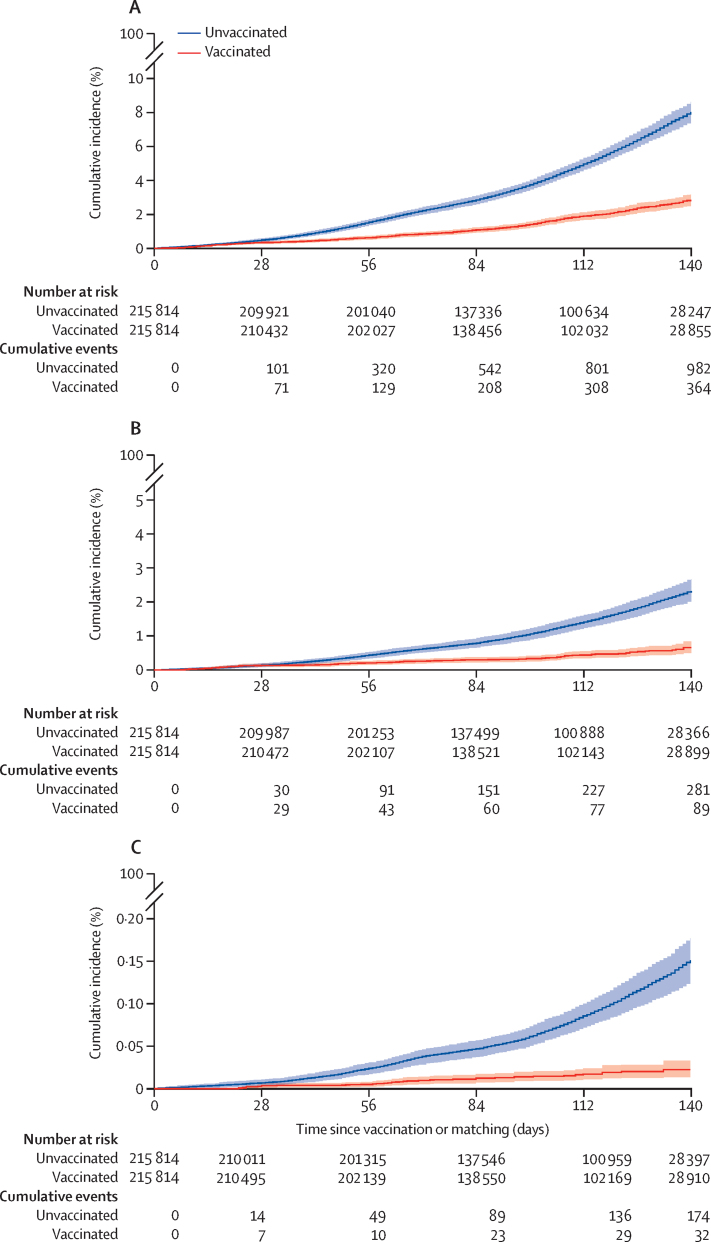
Cumulative incidence of primary COVID-19 outcomes in vaccinated and unvaccinated insured individuals (schemes A and B) by time since vaccination or matching (A) COVID-19-related hospital admissions. (B) COVID-19-related hospital admissions requiring critical or intensive care. (C) COVID-19-related deaths. Solid lines show the cumulative incidence, with shaded areas showing 95% CIs. Number censored at each timepoint is in the appendix (p 17).

Vaccine effectiveness against COVID-19-related death, hospital admission requiring ICU or CCU, and hospital admission 28 days or more after vaccination are shown in table 2. The combined (schemes A and B) cumulative incidence of each of the three primary COVID-19 outcomes in vaccinated and unvaccinated individuals, by time since vaccination or matching, are shown in figure 2. In sensitivity analyses, the vaccine effectiveness for COVID-19-related hospital admissions for the provincial cohort was 68% (95% CI 48–86), which aligned well with the combined results. The cumulative incidence of the three primary COVID-19 outcomes in vaccinated and unvaccinated individuals are shown by scheme and for the Western Cape province in the appendix (pp 19–21). Due to the absence of good quality data on severe hospital admission and insufficient deaths in the Western Cape datatset, we were not able to report on these outcomes for this subcohort.

**Table 2:** COVID-19 event rates and estimated vaccine effectiveness 28 days after vaccination in sub-cohorts compared with the unvaccinated individuals

	**COVID-19-related hospital admission**			**COVID-19-related hospital admission requiring critical or intensive care**			**COVID-19-related death**		
	Vaccinated (events/person-years)	Unvaccinated (events/person-years)	Vaccine effectiveness (95% CI)	Vaccinated (events/person-years)	Unvaccinated (events/person-years)	Vaccine effectiveness (95% CI)	Vaccinated (events/person-years)	Unvaccinated (events/person-years)	Vaccine effectiveness (95% CI)
Scheme A plus B	302/43 770	897/43 452	67% (62–71)	63/43 794	256/43 510	75% (69–82)	28/43 802	163/43 527	83% (75–89)
Scheme A	153/20 128	444/19 773	66% (60–72)	19/20 143	110/19 802	83% (73–90)	11/20 145	75/19 807	85% (75–93)
Scheme B	149/23 462	453/23 679	67% (60–73)	44/23 651	146/23 708	70% (59–79)	17/23 657	88/23 720	80% (69–90)
Western Cape*	12/2654	39/2651	68% (48–86)	..	..	..	..	..	..

* Data on admissions requiring critical or intensive care were not available and too few events occurred to enable analysis of COVID-19-related deaths.

In subgroup analyses, we estimated vaccine effectiveness in various subpopulations according to baseline characteristics for the three main outcomes and in each subcohort (table 3). A similar vaccine effectiveness gradient was seen as in the primary analysis, with vaccine effectiveness estimates against COVID-19-related death generally being highest and COVID-19-related hospital admission generally being lowest across all subpopulations. Vaccine effectiveness was generally well maintained in individuals aged 50 years and older and in individuals with comorbidities. Vaccine effectiveness for the subpopulations of health-care workers with HIV was assessed in scheme B only, because this scheme had the highest number of individuals with HIV. Vaccine effectiveness in health-care workers with HIV was similar to in those without HIV for COVID-19-related hospital admission and hospital admission requiring CCU or ICU admission. Although vaccine effectiveness against COVID-19-related death was seen in health-care works with HIV, this was reduced in comparison with health-care workers without HIV (table 3).

**Table 3:** Estimated vaccine effectiveness 28 days after vaccination in subpopulations according to baseline characteristics versus unvaccinated individuals

		**COVID-19-related hospital admission**			**COVID-19-related hospital admission requiring critical or intensive care**			**COVID-19-related death**		
		Vaccinated (events/person-years)	Unvaccinated (events/person-years)	Vaccine effectiveness (95% CI)	Vaccinated (events/person-years)	Unvaccinated (events/person-years)	Vaccine effectiveness (95% CI)	Vaccinated (events/person-years)	Unvaccinated (events/person-years)	Vaccine effectiveness (95% CI)
**Sex**										
Male										
	Scheme A	39/5452	165/5341	76% (68 to 84)	8/5457	48/5351	82% (68 to 94)	4/5458	32/5353	87% (71 to 98)
	Scheme B	39/4660	84/4666	53% (34 to 69)	11/4661	28/4672	60% (28 to 83)	6/4662	20/4674	71% (38 to 93)
	Western Cape*	4/700	6/700	32% (−36 to 73)	..	..	..	..	..	..
Female										
	Scheme A	113/14 676	280/14 432	60% (51 to 68)	11/14 686	62/14 451	83% (70 to 93)	7/14 688	44/14 454	84% (70 to 95)
	Scheme B	110/18 982	369/19 013	70% (64 to 77)	33/18 990	118/19 036	72% (60 to 82)	11/18 995	68/19 046	83% (71 to 92)
	Western Cape*	10/1954	34/1952	69% (47 to 87)	..	..	..	..	..	..
**Age**										
Age 18–49 years										
	Scheme A	83/15 222	217/14 931	62% (52 to 71)	8/15 231	44/14 946	81% (66 to 94)	2/15 232	24/14 948	90% (73 to 100)
	Scheme B	66/15 612	209/15 962	68% (59 to 76)	14/15 616	55/15 976	73% (56 to 87)	6/15 619	23/15 980	70% (40 to 94)
	Western Cape*	7/1950	27/1948	71% (46 to 90)	..	..	..	..	..	..
≥50 years										
	Scheme A	70/4906	228/4842	66% (57 to 76)	11/4912	67/4856	83% (72 to 93)	9/4913	52/4859	83% (68 to 93)
	Scheme B	83/8030	244/7717	67% (59 to 75)	30/8035	91/7732	68% (53 to 80)	11/8038	65/7740	84% (72 to 93)
	Western Cape*	5/703	13/702	52% (−14 to 81)	..	..	..	..	..	..
**Coexisting risk factors for severe COVID-19**										
None										
	Scheme A	61/13 682	180/13 395	66% (57 to 76)	9/13 689	34/13 407	75% (54 to 91)	5/13 689	18/13 409	72% (40 to 92)
	Scheme B	59/15 283	206/15 328	71% (62 to 79)	20/15 288	61/15 342	67% (49 to 82)	5/15 290	32/15 346	84% (65 to 97)
One or more										
	Scheme A	91/6446	265/6378	66% (57 to 73)	11/6455	77/6394	86% (76 to 94)	6/6456	58/6398	89% (78 to 98)
	Scheme B	90/8359	247/8351	63% (54 to 72)	24/8363	85/8366	71% (57 to 83)	12/8367	56/8374	78% (60 to 89)
**HIV status**										
HIV positive										
	Scheme A	12/997	14/705	..	..	..	..	..	..	..
	Scheme B	18/3802	66/3731	73% (58 to 85)	4/3802	19/3736	79% (51 to 96)	5/3803	15/3738	65% (13 to 93)
No HIV or unknown										
	Scheme A	140/19 131	431/19 068	68% (61 to 73)	140/19 131	431/19 068	85% (76 to 92)	10/19 147	72/19 101	86% (76 to 94)
	Scheme B	131/19 840	387/19 948	66% (59 to 73)	40/19 849	127/19 972	68% (56 to 79)	12/19 854	73/19 982	83% (72 to 97)
**Hypertension status**										
Hypertension present										
	Scheme A	41/2439	150/2416	73% (63 to 81)	5/2443	45/2424	89% (76 to 98)	6/2444	35/2426	84% (68 to 97)
	Scheme B	56/4074	164/4115	66% (55 to 76)	18/4077	62/4125	70% (52 to 85)	9/4080	39/4130	76% (52 to 90)
No hypertension										
	Scheme A	111/17 689	294/17 357	63% (55 to 70)	14/17 700	66/17 377	78% (64 to 89)	6/17 701	40/17 380	86% (72 to 96)
	Scheme B	93/19 568	289/19 564	68% (60 to 75)	26/19 574	84/19 583	69% (55 to 82)	8/19 577	49/19 590	83% (69 to 95)
**No previous documented COVID-19**										
Scheme A		147/17 957	433/17 645	67% (61 to 72)	19/17 972	109/17 672	83% (73 to 90)	10/17 974	67/17 678	86% (75 to 94)
Scheme B		142/21 030	440/21 026	68% (61 to 73)	43/21 038	142/21 055	70% (59 to 80)	16/21 044	86/21 066	81% (70 to 91)
Western Cape*		9/2046	36/2044	73% (52 to 90)	..	..	..	..	..	..

* Data on admissions requiring critical or intensive care were not available and too few events occurred to enable analysis of COVID-19-related deaths; Western Cape data on coexisting clinical risk factors for severe COVID-19 not available.

Using calendar period as proxy, we assessed vaccine effectiveness for two periods when two different dominant viral variants of concern were circulating (table 4). Vaccine effectiveness against COVID-19-related hospital admission and hospital admission requiring critical or intensive care was higher during the delta-dominant period than during the beta-dominant period; however vaccine effectiveness against COVID-19-related deaths was slightly higher during the beta-dominant period than during the delta-dominant period (table 4). Notably, event rates were lower during the beta-dominant wave than during the delta-dominant wave. We did not observe large differences in event rates between vaccinated and unvaccinated individuals in the periods 6–13 and 14–27 days after vaccination or matching. However, we did observe differences at 0–5 days after vaccination or matching, reflective of the so-called healthy vaccine effect during which individuals were symptomatically screened if suspected of having COVID-19 (appendix p 18).

**Table 4:** COVID-19 event rates and estimated vaccine effectiveness 28 days after vaccination or matching during beta variant (B.1.351)-dominant and delta variant (B.1.617.2)-dominant periods

	**COVID-19-related hospital admission**			**COVID-19-related hospital admission requiring critical or intensive care**			**COVID-19-related death**		
	Vaccinated (events/person-years)	Unvaccinated (events/person-years)	Vaccine effectiveness (95% CI)	Vaccinated (events/person-years)	Unvaccinated (events/person-years)	Vaccine effectiveness (95% CI)	Vaccinated (events/person-years)	Unvaccinated (events/person-years)	Vaccine effectiveness (95% CI)
**Scheme A plus B**									
Beta variant-dominant period	33/13 982	89/13 960	62 (42 to 76)	12/13 992	24/13 985	49 (8 to 77)	1/13 996	12/13 991	86 (57 to 100)
Delta variant-dominant period	268/29 788	808/29 492	67 (62 to 71)	51/29 802	232/29 525	78 (71 to 84)	27/29 807	151/29 534	82 (74 to 89)
**Scheme A**									
Beta variant-dominant period	11/5929	29/5919	56 (43 to 68)	0/5931	6/5922	..	0/5931	5/5922	..
Delta variant-dominant period	142/14 199	416/13 854	67 (60 to 72)	19/14 212	105/13 880	82 (72 to 90)	11/14 214	70/13 885	85 (74 to 94)
**Scheme B**									
Beta variant-dominant period	22/8052	61/8041	62 (39 to 79)	12/8061	19/8064	32 (−27 to 73)	1/8064	7/8069	..
Delta variant-dominant period	127/15 590	392/15 638	68 (61 to 74)	32/15 590	127/15 644	75 (64 to 84)	16/15 593	81/15 651	80 (69 to 89)

The beta variant-dominant period was defined as Feb 17 to May 17, 2021, and the delta variant-dominant period as May 18, 2021, until data cutoff (July 17, 2021).

203 viable samples were recovered taken from health-care workers with breakthrough infections (mostly from hospitalised health-care workers) in eight provinces between March 17 and July 17, 2021, and were sued for sequencing. The delta variant was seen in 144 (71%) of 203 samples, the beta variant in 47 (23%), and the alpha (B.1.1.7) variant in six (3%). Other variants were also observed, which were the C.1.2 variant in four (2%) samples and kappa (B.1.617.1), B.1.158, and B.1.1.528 variants in two (1%) samples. We found no indication of increased proportion of any one viral genotype in the breakthrough infections compared with viral variant patterns seen in the national viral genotype surveillance (appendix p 2) Among the vaccinated health-care workers, there were 12 606 breakthrough infections reported as of July 17, 2021, of which 57 (0·5%) were severe and 53 (0·4%) resulted in death (appendix p 10). We found that the majority of severe infections and deaths occurred in individuals aged 50 years and older (appendix p 10).

## Discussion

The Sisonke study, which was conducted during a period when both the delta and the beta variants of concern were circulating in South Africa, supports the real-world effectiveness of the single-dose Ad26.COV2.S COVID-19 vaccine in a large cohort of highly exposed health-care workers, many of whom have HIV. The vaccine was effective against severe outcomes, including COVID-19-related death (83%), COVID-19-related hospital admissions (67%), and COVID-19-related admission to CCUs or ICUs (75%). Most breakthrough infections in these highly exposed health-care workers were asymptomatic or mild, with less than 1% of health-care workers having a severe SARS-CoV-2 infection that resulted in hospitalisation or death.

This study was conducted during South Africa's third and most deadly COVID-19 wave, and during the transition in dominance from one variant of concern (beta) to another (delta) up to 5 months after vaccination. South Africa has had an important role in the initial assessment of the Ad26.COV2.S phase 3 efficacy trial, ENSEMBLE.^4^ The ENSEMBLE trial found a moderate reduction in vaccine efficacy (64% efficacy for moderate-to-severe or critical COVID-19 and 81·7% for severe or critical COVID-19) for South African participants compared with US participants, which was attributed to the beta variant circulating in South Africa while other variants were circulating in other countries. However, these results gave sufficient confidence to allow administration of the single-dose Ad26.COV2.S vaccine to health-care workers before the South African national roll-out started (on May 17, 2021), ahead of the expected third wave. Our study, which started before licensure of the vaccine in South Africa, validates that decision and we found that vaccine effectiveness is upheld for clinically important endpoints during surges when morbidity and mortality are severely affected by restricted health system capacity, in particular ICU services,^11^, ^12^ and despite the emergence of a new variant of concern.

Health-care workers are at increased risk of SARS-CoV-2 infection and have been highly affected worldwide. Our findings are similar to those of other field evaluations in cohorts of health-care workers with different COVID-19 vaccines and viral variants.^13^, ^14^, ^15^ South Africa is a country with a large burden of comorbidities and the majority of health-care workers who died due to COVID-19 had at least one comorbidity and many had multiple comorbidities. The Sisonke study also provides the first reassurance that this vaccine protects people with HIV, information that is much needed in a global context where fewer than 2500 people with HIV have participated in published efficacy trials.^16^, ^17^

Smaller real-world effectiveness studies investigating Ad26.COV2.S have been conducted in other regions of the world and largely support our findings.^18^, ^19^, ^20^, ^21^, ^22^, ^23^ A study in the Netherlands assessing the vaccine effectiveness of Ad26.COV.2 against hospitalisation and ICU admission in the general population showed high protection of 91%.^24^ Our vaccine effectiveness results are lower than in this report, which might be due to several reasons. First, vaccination in the Sisonke study occurred during the downturn of the beta-dominant second wave and the commencement of the delta-dominant third wave in the region; and second, our study was conducted in highly exposed health-care workers with multiple comorbidities. The beta variant of SARS-CoV-2 has been shown to affect vaccine effectiveness, which could have affected our estimates. The high prevalence of comorbidities, including a high HIV prevalence in one of the schemes, could have reduced vaccine effectiveness compared with other studies.

To robustly assess vaccine effectiveness, we did three analyses using datasets from two medical insurance schemes and a provincial public sector database of health-care workers. Although schemes A and B allowed us to make comparisons with matched working individuals (who might or might not have been health-care workers), SARS-CoV-2 exposure might be lower in the matched population than in the health-care worker population. The provincial dataset allowed us to compare vaccinated and unvaccinated health-care workers in a sensitivity analysis to address this limitation and we found very similar vaccine effectiveness estimates in these analyses. The large and comprehensive datasets enabled high rates of matching, optimising generalisability of our findings. The overall size of the study has enabled good precision for most primary outcomes. Although we found a range of vaccine effectiveness estimates from the different datasets, we are reassured that the estimates are consistent. Linkage to the EVDS for comparison groups minimised misclassification of follow-up time, and linkage to the death registry endpoint minimised ascertainment bias.

We did not investigate vaccine effectiveness for overall infection with SARS-CoV-2 because, unlike the primary outcomes, vaccine effectiveness against infection is largely driven by differing access to testing and because many people would not go into hospital for a mild infection, use of claims databases to track infections would not be as effective as for admissions. This variability in available information could result from either testing in the public sector, through workplace testing programmes, or out-of-pocket payment for testing. Many reasons exist for changing and varied testing patterns over time and throughout the country, including prioritisation of testing in some provinces during the third wave due to restricted capacity. Our assessment of vaccine effectiveness in health-care workers who have been admitted to hospital, the ICU or CCU, or who died are less affected by differences in testing behaviour than among non-hospitalised health-care workers with SARS-CoV-2 infection, because most people who are admitted to hospital are tested. Likewise, most deaths occurred in people during or after a stay in hospital.

Health-care workers in South Africa are predominantly female and middle-aged (ie, aged 40–60 years) and so our matched population was also predominantly female and middle-aged, restricting the number of men and older people included in our study. However, subgroup analyses confirmed similar protection in men and older people. Although the quality of diagnostic PCR testing has been carefully controlled in South Africa and antigen testing was allowed since October, 2021, our vaccine effectiveness estimates might also have been affected by imperfect sensitivity or specificity of these tests. Other differences in the datasets, such as presence and number of comorbidities, age, and HIV prevalence, might have contributed to differences in vaccine effectiveness.

Limitations of our study include the possibility of selection bias due to linking of data via medical schemes. Although the health-care workers and essential workers in scheme B were matched by their exposure risk, exposure could have differed as well as their health-seeking behaviour. Because this was not a randomised trial, matching might not have completely removed residual confounders or bias.

Our study has important policy ramifications, especially for the sub-Saharan region, which has faced three variants of concern in quick succession, constrained access to effective vaccines against these variants, and logistical difficulties in rapidly scaling up delivery. We found that a single-dose vaccine provided good protection within 2–5 months after vaccination and this effectiveness was maintained with the emergence of a second variant of concern. Ad26.COV2.S remains an important vaccine in settings where alternative regimens impose cold-chain logistics or require people in remote areas or dependent on daily paid work to return for a second vaccination within a short timeframe. Single-dose regimens also offer an opportunity to move quickly and efficiently to protect susceptible populations. Real-world effectiveness studies have shown the loss of effectiveness of COVID-19 vaccines over time. This loss in effectiveness could be attributed to waning immunity or the emergence of a new variant of concern. The Sisonke study will add critical information to the durability of the single-dose regimen. The recent addition of a booster to the Sisonke study, per a protocol amendment on Oct 25, 2021, will provide critical information on effectiveness of booster doses administered from 6 to 9 months after initial vaccination. For the world's most unvaccinated region, this single-dose vaccine provides a robust, practical, and effective emergency solution to mitigate the worst effects of COVID-19.

## Data sharing

Individual participant data will not be made available. Study protocol, statistical analysis plan, and analytical code will be available from the time of publication in response to any reasonable request to the corresponding author.

## Declaration of interests

L-GB declares honoraria for advisory roles from MSD, ViiV Health Care, and Gilead. RJL declares Department of Science and Innovation and South African Medical Research Council (SAMRC) funding to the KwaZulu-Natal Research Innovation and Sequencing Platform at the University of KwaZulu-Natal for the Network for Genomic Surveillance South Africa, which supported the genomic sequencing for this study; and committee membership of the Ministerial Advisory Committee on COVID-19 Vaccines (a committee that makes recommendations to the Minister of South Africa on the national COVID-19 vaccine programme. CC declares grants or contracts from CDC, PATH, the Bill & Melinda Gates Foundation, SAMRC, Wellcome Trust, and Sanofi Pasteur in the past 36 months. MG reports grants from SAMRC during the conduct of the study, and grants from the Bill & Melinda Gates Foundation outside of the submitted work. DBa reports grants from US National Institutes of Health (NIH) and Janssen during the conduct of the study; grants from Defense Advanced Research projects Agency, Massachusetts Consortium on Pathogen Readiness, Ragon Institute, the Bill & Melinda Gates Foundation, SAMRC, Henry Jackson Foundation, Musk Foundation, Gilead, Legend Bio, CureVac, Sanofi, Intima Bio, Alkermes, and Zentalis; and personal fees from SZQ Bio, Pfizer, Celsion, Avidea, Laronde, Meissa, and Vector Sciences outside of the submitted work DBr has three patents (63/121,482; 63/133,969; 63/135,182) licensed to Janssen. All other authors declare no competing interests.
