# Supplementary material for: Effectiveness of the Ad26.COV2.S vaccine in health-care workers in South Africa (the Sisonke study): results from a single-arm, open-label, phase 3B, implementation study
Source: Lancet. 2022 Mar 19;399(10330):1141–53. doi: 10.1016/S0140-6736(22)00007-1 (PMC8930006; doi:10.1016/S0140-6736(22)00007-1)
Supplement: Supplementary appendix [file mmc1.pdf]

THE LANCET

Supplementary appendix

This appendix formed part of the original submission and has been peer reviewed. We post it as supplied by the authors.

Supplement to: Bekker L-G, Garrett N, Goga A, et al. Effectiveness of the Ad26.COV2.S vaccine in health-care workers in South Africa (the Sisonke study): results from a single-arm, open-label, phase 3B, implementation study. *Lancet* 2022; **399:** 1141–53.

SUPPLEMENT.

Contents:

Figure S1: Viral lineage patterns in South Africa demonstrating shift from Beta to Delta dominance (2a) and in breakthrough infections in Sisonke participants (2b) 2

Table S1: Data Sources 3

Table S2: Variable Definitions 5

Table S3: Breakthrough Infections in all Sisonke vaccine recipients according to WHO severity classification 9

Figure S2: Flow diagram showing person-level data on vaccinated recipients and unvaccinated controls in the Western Cape provincial dataset available for analysis 10

Table S4: Baseline demographic and clinical characteristics of population eligible for sampling and those included in analysis (Scheme A) 11

Table S5: Baseline demographic and clinical characteristics of population eligible for sampling and those included in analysis (Scheme B) (to be updated) 13

Table S6: Baseline demographic and clinical characteristics of population eligible for sampling and those included in analysis (Western Cape) 15

Figure S3: Covariate Balance (Love) Plots showing the standardized difference in means between vaccinated and unvaccinated groups for different CDC risk criteria 16

Table S7: COVID-19 event rates and estimated vaccine effectiveness 0-5 days, 6-13 days and 14-27 days after vaccination in scheme participants 17

Figure S4a: Cumulative incidence of different Covid-19 outcomes in vaccinated and unvaccinated individuals: Scheme A 19

Figure S4b: Cumulative incidence of different Covid-19 outcomes in vaccinated and unvaccinated individuals: Scheme B 20

Figure S4c: Cumulative incidence of Covid-19 related hospitalisation and death in vaccinated and unvaccinated individuals: Western Cape cohort 21

Figure S1a: Viral lineage patterns in South Africa demonstrating shift from Beta to Delta dominance (N=7005)

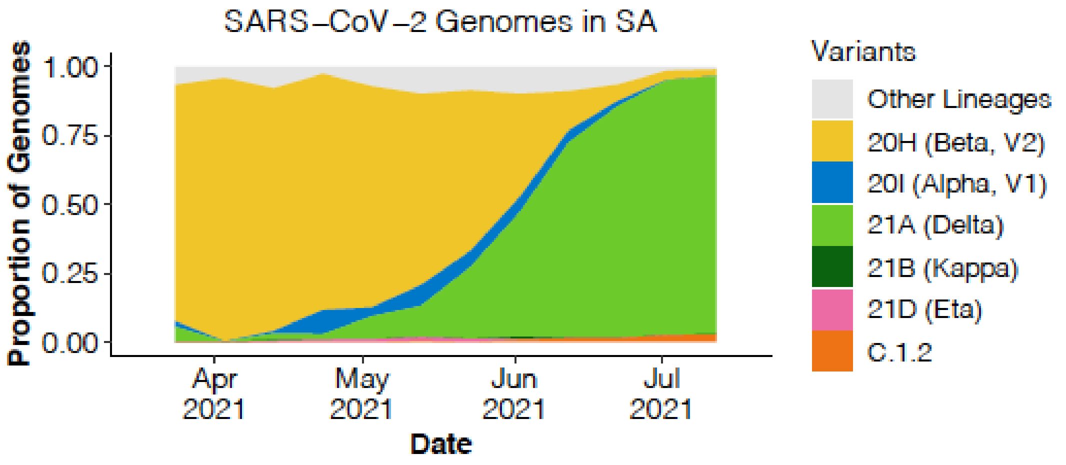

Figure S1b: Viral lineage patterns seen in breakthrough infections in Sisonke participants (n=203)

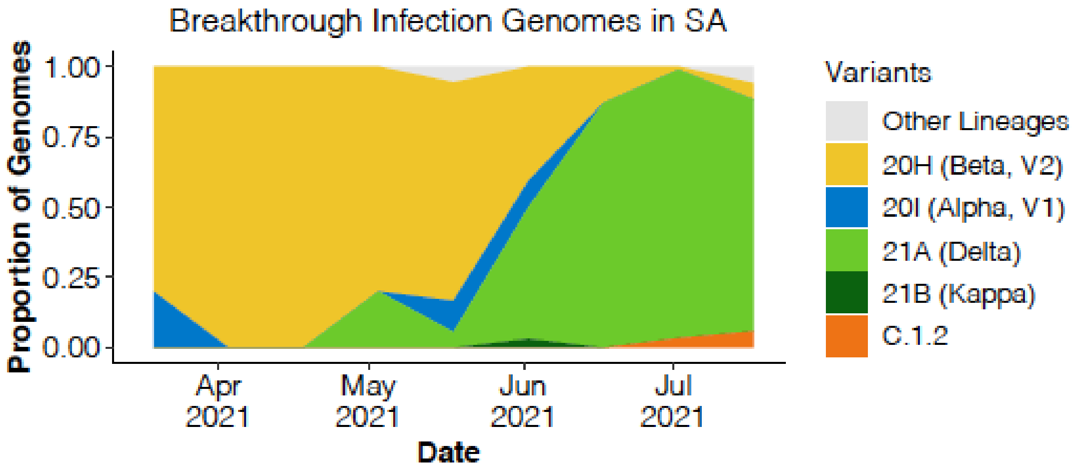

Figure S1c: A graph depicting the 7-day moving average number of SARS-CoV-2 infections in South Africa since the start of the pandemic and the daily vaccination numbers for Sisonke 3B study

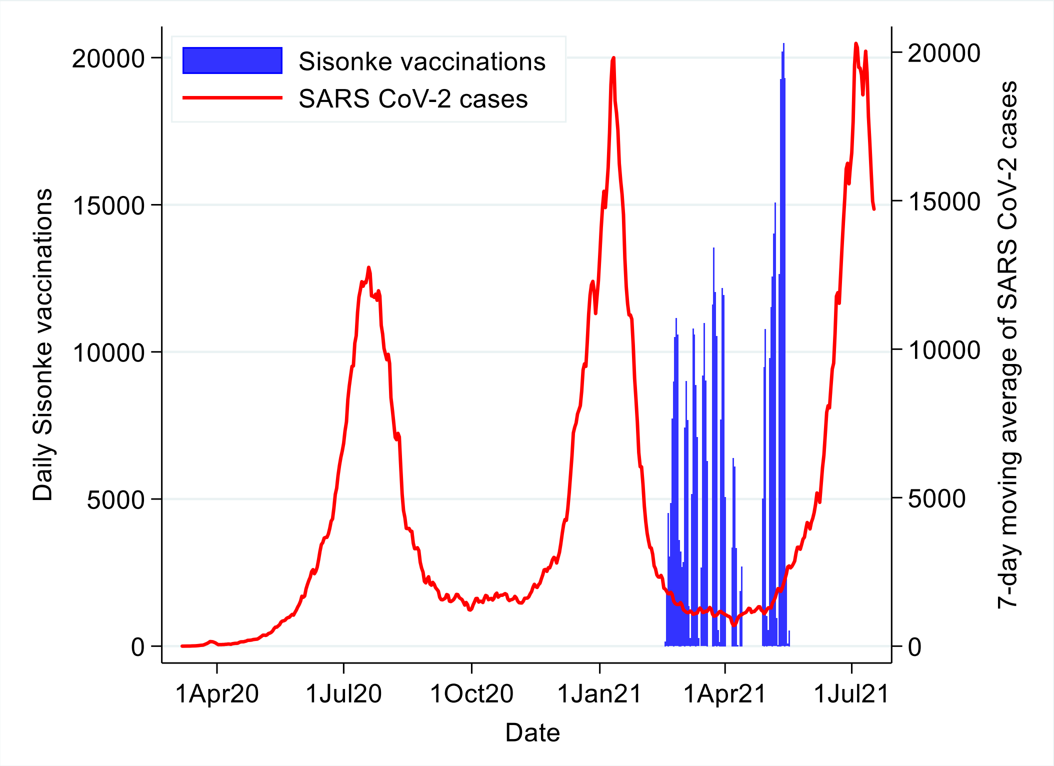

Note a two week gap in vaccinations (13 to 27 April) following the FDA and subsequent SAHPRA pause in Ad26·COV2·S vaccinations

Table S1: Data sources

Name	Description
1. Electronic Vaccination Data System (EVDS)	All people who receive a Covid-19 vaccination in South Africa are required to enter their South African civil identification number (ID number) or passport number, age and gender onto an electronic vaccine data portal (https://vaccine.enroll.health.gov.za/#/). Vaccine type, lot number, site identifier and date of vaccination are captured at the time of administration. The EVDS system was initiated at the start of the Sisonke study and captured the details of all HCWs who received a vaccine as part of the study. It also contributed vaccination status and date of vaccination for matched counterparts for the comparative analyses of vaccine effectiveness.
2. Sisonke Desk	The Sisonke Desk is staffed by a team of safety physicians and records all instances of reactogenicity, vaccine adverse events and breakthrough infections reported by participants or health providers. It is accessible through a toll-free hotline, online self-administered data collection tool or email address, details for which were included in consent forms and widely advertised in take-home information sheets post vaccination and social media. It is also enriched through weekly linkage with the South African Vital Register (Source 3), NMCSS (Source 4) and DATCOV (Source 7). This allowed comprehensive passive surveillance of all hospitalisations, deaths and breakthrough infections. Participants who self-reported, or were identified through weekly linkages, were followed up with text messages, telephonic interviews with themselves, family members or health providers and review of medical records.
3. National Population Register	The South African National Population Vital Register records details of all deaths registered throughout the country and is held by the National Department of Home Affairs. A research equivalent, which details fact, date and site of death, is curated by the South African Medical Research Council (SAMRC), updated weekly and has been used to track excess mortality throughout the pandemic[1].
4. Covid-19 Notifiable Medical Conditions Sentinel Surveillance (NMCSS) master list	Covid-19 is a notifiable condition in South Africa and all laboratories and healthcare providers are required to report all PCR or antigen tested confirmed cases to the National Institute for Communicable Diseases (NICD). This is used to compile a Covid-19 Notifiable Medical Conditions Sentinel Surveillance (NMCSS) master list. This master list was used for passive surveillance of the Sisonke vaccine recipients (Source 2). Efforts to link this to medical schemes are underway but had not been completed by the time of this analysis.
5. Scheme A and Scheme B	Approximately 8.9 million South Africans are members of private healthcare funds (referred to as ‘medical schemes’ in South Africa), which by law offers a minimum package of prescribed minimum benefits[2]. Membership to a medical scheme is voluntary, and membership to some medical scheme funds are employer based, whilst others are open to any individual and any employer. The healthcare delivery system in South Africa is divided between private providers of healthcare (funded by medical schemes or out-of-pocket) and public facilities (funded primarily by general taxation). The organisations (Referred to as “Scheme A” and

	"Scheme B") used in these analyses are administration and managed care services to medical schemes. Public sector workers include health workers employed by the State, as well as many workers classified as essential workers by the Covid-19 National Disaster Management Act. These workers were required to perform their duties from their place of work throughout the pandemic during periods of restricted movement and are deemed highly exposed to SARS-CoV-2.<br>Both 'Schemes' have rich longitudinal demographic, clinical and claims data on thier members. Covid-19 claims data includes tests and detailed information on hospital admissions, including categorisation as to whether these were for symptomatic Covid-19.
6. Western Cape Provincial Health Data Centre	The Western Cape's Provincial Health Data Centre (PHDC) is the largest (8 million unique people active in the past decade) and most comprehensive public health sector database in sub-Saharan Africa.[3,4] Established in 2015, it integrates data from 22 individual sources (e.g. hospitalisations, pharmacy systems, laboratory and disease registers) to generate a longitudinal record for all people accessing the public health system in the province. As Covid-19 is a notifiable medical condition with statutory requirements for reporting to government, the PHDC has included Covid-19-related clinical data for the whole province, including recipients of care in the private sector, estimated to be used by approximately 30% of the provincial population, and a higher proportion of HCWs. Data on all state-employed health care workers are linkable to the PHDC in a secure environment to facilitate automated aggregate reporting of Covid-19 HCW infections, hospitalisations and deaths.
7. DATCOV	The DATCOV database, maintained by the NICD, set up at the onset of the Covid-19 pandemic contains data on hospitalised individuals from all public and private hospitals with a positive real-time SARS-CoV-2 reverse transcription polymerase chain reaction (rRT-PCR) assay or a positive SARS-CoV-2 antigen test, regardless of age or reason for admission. This includes patients who had Covid-19 symptoms, were admitted for isolation, acquired nosocomial Covid-19 infection, or tested positive incidentally.

1. Bradshaw D, Dorrington RE, Laubscher R, Moultrie TA, Groenewald P. Tracking mortality in near to real time provides essential information about the impact of the COVID-19 pandemic in South Africa in 2020. South African Medical Journal. 2021 Aug 1;111(8):732-40.
2. Council for Medical Schemes annual report 2019/2020, 2021
3. Western Cape Department of Health in collaboration with the National Institute for Communicable Diseases, S.A., *Risk factors for COVID-19 death in a population cohort study from the Western Cape Province, South Africa.* Clin Infect Dis, 2020.
4. Boulle, A., et al., *Data Centre Profile: The Provincial Health Data Centre of the Western Cape Province, South Africa.* Int J Popul Data Sci, 2019. **4**(2): p. 1143.

Table S2: Variable Definitions

Variable	Definition
**Outcomes**	
Covid-19 related hospitalization	Hospitalisations were defined by availability of information available in the source data. Scheme A considered admissions to be Covid-19 related if they occurred within 14 days of a positive PCR test result and the admission was likely to be Covid-19 related (e.g. respiratory distress, pneumonia, pulmonary embolism), or if the primary reason for admission was Covid-19 (ICD code U07.1). For Scheme B a hospitalisation was defined as Covid-19 related if the primary reason for admission was Covid-19 (ICD code U07.1) as submitted by the attending physician. In the Western Cape PHDC, a hospitalisation was considered Covid-19 related if the date of admission occurred 14 days before or 9 days after a positive Covid-19 PCR or antigen test. In all datasets the date the person was first admitted to hospital during the follow-up period was used.
Covid-19 related hospitalization requiring critical or intensive care	Hospitalisations considered Covid-19 related during which the person was provided critical care (usually for high flow oxygen) or intensive care services (usually for mechanical ventilation). The date the person was first admitted to hospital during the follow-up period was used, even if the person was later transferred to critical or intensive care.
Covid-19 related death	A death in an individual where non-natural cause of death was ruled out and which met one of the following criteria:<br>• During a Covid-19 hospitalisation<br>• Within 14 days of discharge from hospital for Covid-19<br>• Within 28 days of a positive Covid-19 PCR or antigen test in persons who were not hospitalised during that interval<br>• Within 28 days of positive Covid-19 PCR or antigen test in persons who were admitted to hospital for respiratory or other reasons associated with severe Covid-19 but whose hospitalisation was not identified to the scheme as a Covid-19 admission (following individual adjudication blinded by vaccination status).
**Matching variables**	
Age	Age in completed years at the time of vaccination with the Ad26.COV2.S vaccine as part of the Sisonke study. For controls, the date of the vaccination of the intervention participant was used to calculate age at last birthday. Two-year age bands were used for matching in both schemes, and a less than five-year age difference for the Western Cape PHDC analysis.
Sex	Male or female as defined in the dataset.
Risk factors for severe Covid-19	Risk factors for severe Covid-19 were adapted from South Africa's list of Prescribed Minimum Benefits Chronic Disease List (CDL) and the Centres for Disease Control and Prevention (CDC) list of conditions associated with increased risk of severe Covid-19[1]. The sum of risk factors was used. Four categories were defined:<br>• None<br>• 1<br>• 2<br>• 3 or more<br>Clinical risk factors were not available in the Western Cape PHDC.

**Variable**	**Definition**
Geographical location (health district)	Magisterial district information was available in both schemes (several hundred per scheme) and was mapped to South Africa’s 52 health districts using a geo-location classification previously used in malaria studies. Geographical location was not used as a matching criterion for the Western Cape PHDC.
Socio-economic status	Level of income was available for members of Scheme B as it is a closed employer-based scheme. Four categories were defined based on monthly income:<br>• Low Income (<ZAR18 000 per month)<br>• Lower (ZAR18 000 – R21 999 per month)<br>• Middle (ZAR22 000 – R27 999 per month)<br>• Upper (>ZAR28 000 per month)<br>Scheme A did not have access to income level, and so used plan option as a proxy for socio-economic status, categorised into three groups based on ZAR value of monthly contributions:<br>• Low (<ZAR1600)<br>• Middle (ZAR 1600-2350)<br>• Upper (>ZAR 2350)<br>A socio-economic status variable was not used as a matching criterion for the Western Cape PHDC.
Prior documented SARS-Cov-2 infection	This was classified based on evidence of a positive PCR or antigen test in the period prior to vaccination or matching. Four categories were defined:<br>• None<br>• Most recent infection <90 days ago<br>• Most recent infection 90-179 days ago<br>• Most recent infection ≥ 180 days ago<br>Testing rates varied substantially between the datasets, ranging from very low (Scheme B) to higher (PHDC). The testing rate in Scheme B was very low, and consequently was not used as a matching criterion as otherwise too many Sisonke vaccines could not be matched to a counterpart.
Number of negative Covid-19 tests	Health workers in the provincial dataset had priority access to Covid-19 testing and were frequently tested to limit duration of quarantine and relieve staff shortages. The number of Covid-19 tests prior to the start of the Sisonke study were used as a proxy of care- and test-seeking behaviour in this cohort. Five categories were defined:<br>• None<br>• 1-2<br>• ≥3
Occupational group	This was available for the provincial dataset and was classified broadly based on level of occupational exposure to Covid-19. Six categories were defined:<br>• Nurses<br>• Management and support (including management, administrative, maintenance and technical/ scientific staff)<br>• Ancillary (for example cleaners, porters and laundry staff)<br>• Doctors<br>• Allied health services and dental<br>• Emergency medical services
**Other variables**	

Variable	Definition
Documented SARS-CoV2-Infection	PCR-confirmed infection. If multiple positive PCR tests were available, the date of the first positive PCR test was used.
Cancer	Claim(s) submitted for cancer treatment in 2021 (Scheme B) or in the 12-month period preceding data of vaccination or matching (Scheme A).
Cardiovascular disease	• Cardiac failure<br>• Cardiomyopathy<br>• Coronary artery disease<br>• Dysrhythmias<br>• Peripheral arterial disease<br>• Cerebrovascular disease (including stroke)
Chronic renal disease	Chronic renal disease
Chronic respiratory disease	• Asthma<br>• COPD<br>• Bronchiectasis
Diabetes mellitus	• Diabetes Mellitus Type 1<br>• Diabetes Mellitus Type 2
HIV	HIV
Hypertension	Hypertension
Liver disease	• Alcoholic liver disease<br>• Fatty liver disease<br>• Cirrhosis
Neurological disorders	• Epilepsy<br>• Parkinson’s disease<br>• Dementia (any cause, including Alzheimer’s disease)
Overweight / obesity	Body mass index >25
Severe mental disorders	• Bipolar mood disorder<br>• Schizophrenia<br>• Alcohol and substance misuse
Sickle cell disease or thalassemia	• Sickle cell disease<br>• Thalassemia
District	Health district (n=52) in which individual resides
Breakthrough infections	Any PCR SARS-CoV-2 documented infection occurring more than 28 days after vaccination.
Mild, moderate and severe Covid-19	This was based on the World Health Organisation’s Clinical Progression Scale with adaptations to align with the local South African context such as the lack of availability of hospital or intensive care unit beds. Covid-19 infection outcomes were defined: 1) mild disease: asymptomatic Covid-19 infection and symptomatic disease managed at home with no oxygen therapy or hospitalisation less than 24 hours or for non Covid-19 reasons; 2) Moderate disease: hospital admission and care for more than 24 hours or oxygen therapy provided by nasal prongs or face mask; 3) Severe disease requiring more intensive care with high flow oxygen therapy or non–invasive ventilation or mechanical ventilation or requiring vasopressors dialysis or extracorporeal membrane oxygenation; and 4) any breakthrough infection that resulted in death.

Variable	Definition
Beta wave infections	All infections recorded between start of the study (17 February 2021) and 15 May 2021. This included the second wave in South Africa and the first part of the third wave.
Delta wave infections	All infections recorded between 15 May and end of the study on 17 July 2021.

1. Centers for Disease Control and Prevention. COVID-19. August 2021. Accessed 31 August 2021. https://www.cdc.gov/coronavirus/2019-ncov/need-extra-precautions/people-with-medical-conditions.html

Table S3: Breakthrough Infections in all Sisonke vaccine recipients according to WHO severity classification

	Mild disease (N=10871)	Moderate disease (N=351)	Severe disease (N=57)	Death (N=53)	Unable to trace (N=1274)	Total (N=12 606)
**Age group (years) – no. (%)**						
18-19	30 (0·3)	0 (0)	0 (0)	0 (0)	1 (0·1)	**31**
20-29	1577 (14·5)	10 (2·8)	0 (0)	1 (1·9)	142 (11·1)	**1730**
30-39	2937 (27)	51 (14·5)	6 (10·5)	2 (3·8)	346 (27·2)	**3342**
40-49	3071 (28·2)	90 (25·6)	13 (22·8)	9 (17)	374 (29·4)	**3557**
50-59	2260 (20·8)	120 (34·2)	24 (42·1)	25 (47·2)	306 (24)	**2735**
60-69	814 (7·5)	59 (16·8)	10 (17·5)	10 (18·9)	93 (7·3)	**986**
70-79	169 (1·6)	20 (5·7)	2 (3·5)	4 (7·5)	8 (0·6)	**203**
>80-89	13 (0·1)	1 (0·3)	2 (3·5)	2 (3·8)	4 (0·2)	**22**
**Sex– no. (%)**						
Female	8293 (76·3)	249 (70·9)	37 (64·9)	33 (62·3)	955 (75·0)	**9567**
Male	2578 (23·7)	102 (29·1)	20 (35·1)	20 (37·7)	319 (25·0)	**3039**
**Admission status* – no. (%)**						
Inpatient	12 (0·1)	240 (68·4)	57 (100)	45 (84·9)	0 (0)	**354**
Outpatient	10859 (99·9)	111 (31·6)	0 (0)	8 (15·1)	57 (4·6)	**11035**
Indeterminable	-	-	-	-	1217 (95·5)	**1217**

*27 participants have missing data; ** Unable to contact participant. However, these participants are assumed to be asymptomatic/mild/moderate infection as they were not found in the national Covid-19 hospitalisation database (DATCOV) or the National Population Register (for deaths).

Figure S2: Flow diagram showing person-level data on vaccinated and unvaccinated controls in the Western Cape provincial dataset available for analysis

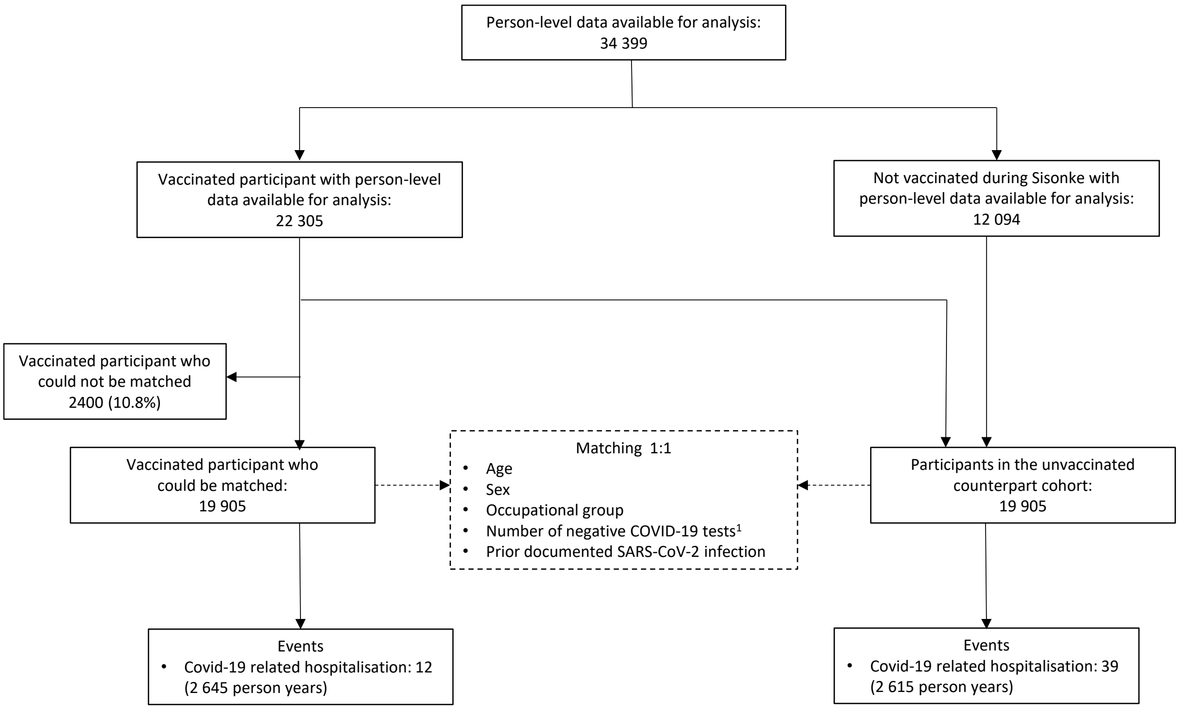

1. Before 17 February 2021

Table S4: Sisonke vaccine recipients who are eligible for matching and successfully matched, by matching variables (Scheme A)

Characteristic	Sisonke recipients	
	Eligible (N= 100 826)	Matched (N=99 084)
Median age (IQR), years	40 (32, 50)	39·7 (31·6, 49·2)
**Sex – no. (%)**		
Female	73393 (72·8)	72093 (72·8)
Male	27433 (27·2)	26992 (27·2)
**Age groups (years) – no. (%)**		
18-39	48125 (47·7)	47430 (47·9)
40-49	26932 (26·7)	26499 (26·7)
50-59	16904 (16·8)	16505 (16·7)
60-69	7159 (7·1)	6988 (7·1)
70-79	1529 (1·5)	1492 (1·5)
≥80	177 (0·2)	170 (0·2)
**Plan Option (as proxy for socio-economic status) – no.(%)**		
High	40048 (39·7)	39282 (39·6)
Middle	36465 (36·2)	35828 (36·2)
Low	24313 (24·1)	23975 (24·2)
**Geographical location – no. (%)**		
Eastern Cape	5852 (5·8)	5671 (5·7)
Free State	3861 (3·8)	3672 (3·7)
Gauteng	42317 (42·0)	42136 (42·5)
KwaZulu-Natal	16608 (16·5)	16152 (16·3)
Limpopo	2058 (2·0)	1955 (2·0)
Mpumalanga	2381 (2·4)	2283 (2·3)
NorthWest	2345 (2·3)	2253 (2·3)
Northern Cape	925 (0·9)	834 (0·8)
Western Cape	23786 (23·6)	23451 (23·7)
Unallocated	693 (0·7)	678 (0·7)
**Number of risk factors for severe COVID-19 – no.(%)**		
0	67398 (66·8)	66741 (67·4)
1	25087 (24·9)	24495 (24·7)
2	6645 (6·6)	6200 (6·3)
3+	1696 (1·7)	1649 (1·7)

Characteristic	Sisonke recipients	
	**Eligible (N= 100 826)**	**Matched (N=99 084)**
**CDC risk factors – no. (%)**		
Diabetes mellitus	4427 (4·4)	4207 (4·2)
Hypertension	12951 (12·8)	12421 (12·5)
HIV	4919 (4·9)	4720 (4·8)
Cardiovascular disease	1405 (1·4)	1343 (1·4)
Chronic liver disease	295 (0·3)	280 (0·3)
Chronic renal disease	160 (0·2)	154 (0·2)
Cancer	944 (0·9)	909 (0·9)
Chronic Respiratory Disease	5446 (5·4)	5244 (5·3)
Neurological Disorders	904 (0·9)	870 (0·9)
Overweight/ obesity	10287 (10·2)	10080 (10·2)
Severe mental disorders	2039 (2·0)	1958 (2·0)
Solid organ transplant recipient	30 (<0·1)	30 (<0·1)
**COVID-19 history – no. (%)** *		
Ever tested for COVID-19	88838 (88·1)	88691 (89·5)
Documented prior COVID-19 infection (Wave 1) *	5477 (5·4)	5616 (5·7)
Documented prior COVID-19 infection (Wave 2)	6511 (6·5)	4777 (4·8)

* Infection before 1 October 2020

Table S5: Sisonke vaccine recipients who are eligible for matching and successfully matched, by matching variables (Scheme B)

Characteristic	Sisonke recipients	
	Eligible (N= 123 200)	Matched (N=116 729)
Median age (IQR), years	45 (38, 53)	45 (38,53)
**Sex – no. (%)**		
Female	98138 (79·7)	93359 (80·0)
Male	25062 (20·3)	23370 (20·0)
**Age groups (years) – no. (%)**		
18-39	37778 (30·7)	33906 (29·0)
40-49	42530 (34·5)	41247 (35·3)
50-59	33315 (27·0)	32473 (27·8)
60-69	9110 (7·4)	8690 (7·4)
70-79	433 (0·4)	383 (0·3)
≥80 yr	34 (<0·1)	30 (<0·1)
**Income level - no· (%)**		
Low Income	65120 (52·9)	63500 (54·4)
Lower	18515 (15·0)	17747 (15·2)
Middle	13002 (10·6)	11903 (10·2)
Upper	26563 (21·6)	23579 (20·2)
**Geographical location – no. (%)**		
Eastern Cape	15419 (12·5)	14492 (12·4)
Free State	8042 (6·5)	7604 (6·5)
Gauteng	22907 (18·6)	22354 (19·2)
KwaZulu-Natal	27360 (22·2)	25401 (21·8)
Limpopo	14738 (12·0)	14096 (12·1)
Mpumalanga	7090 (5·8)	6832 (5·9)
North West	8664 (7·0)	8397 (7·2)
Northern Cape	2465 (2·0)	2165 (1·9)
Western Cape	16515 (13·4)	15388 (13·2)

Characteristic	Sisonke recipients	
	**Eligible (N= 123 200)**	**Matched (N=116 729)**
**Number of risk factors for severe COVID-19 – no. (%)**		
0	78507 (63·7)	74951 (64·2)
1	31230 (25·3)	30053 (25·7)
2	10822 (8·8)	9841 (8·4)
3+	2641 (2·1)	1884 (1·6)
**CDC risk factors – no. (%)**		
Diabetes mellitus	9959 (8·1)	8805 (7·5)
Hypertension	22192 (18·0)	20347 (17·4)
HIV	20194 (16·4)	19032 (16·3)
Cardiovascular disease	1223 (1·0)	1017 (0·9)
Chronic liver disease	2 (<0·1)	2 (<0·1)
Chronic renal disease	166 (0·1)	124 (0·1)
Cancer	840 (0·7)	764 (0·7)
Chronic respiratory disease	3129 (2·5)	2661 (2·3)
Neurological disorders	727 (0·6)	593 (0·5)
Overweight/ obese	1140 (0·9)	978 (0·8)
Severe mental disorders	903 (0·7)	742 (0·6)
**COVID-19 history – no. (%)** *		
Ever tested for COVID-19	43518 (35·3)	40964 (35·1)
None	109335 (88·7)	103600 (88·8)
< 90 days ago	2526 (2·1)	2406 (2·1)
90-179 days ago	2904 (2·4)	2709 (2·3)
≥ 180 days ago	8435 (6·8)	8014 (6·9)

* Before date of vaccination/matching

* Infection before 1 October 2020; prior COVID-19 infection not used as a matching variable

Table S6: Sisonke vaccine recipients who are eligible for matching and successfully matched, by matching variables (Western Cape)

Characteristic	Sisonke vaccine recipients	
	Eligible (N=22 305)	Matched (N=19 950)
**Sex - no·(%)**		
Female	16219 (72·7)	14641 (73·4)
Male	6086 (27·3)	5310 (26·6)
**Age groups (years) – no. (%)**		
18-39	9980 (44·7)	9089 (45·6)
40-49	6197 (27·8)	5489 (27·5)
50-59	5225 (23·4)	4593 (23·0)
60-69	903 (4·0)	780 (3·9)
70-79	0 (0·0)	0 (0·0)
≥80	0 (0·0)	0 (0·0)
**Median age (IQR), years**	41 (33,68)	41 (24,61)
**Occupational group – no. (%)**		
Nurses	9334 (41·8)	8483 (42·5)
Management and support	4227 (19·0)	4010 (20·1)
Ancillary	3094 (13·9)	2905 (14·6)
Allied health services, dental	1777 (8·0)	1514 (7·6)
Emergency medical services	1279 (5·7)	1101 (5·5)
**Number of previous negative tests*– no. (%)**		
0	11579 (51·9)	10623 (53·2)
1	6196 (27·8)	5500 (27·6)
2	2714 (12·2)	2322 (11·6)
≥ 3	1816 (8·1)	1506 (7·5)
**Previous documented COVID-19 infection**– no. (%)**		
None	16573 (74·3)	15229 (76·3)
< 90 days ago	1613 (7·2)	1382 (6·9)
90-179 days ago	1095 (4·9)	762 (3·8)
≥ 180 days ago	3024 (13·6)	2578 (12·9)

* Before 17 Feb 2021
** Before date of vaccination/matching

Figure S3: Covariate balance (love) plots showing the standardized difference in means between vaccinated and unvaccinated groups for the different CDC risk criteria

A: Scheme A

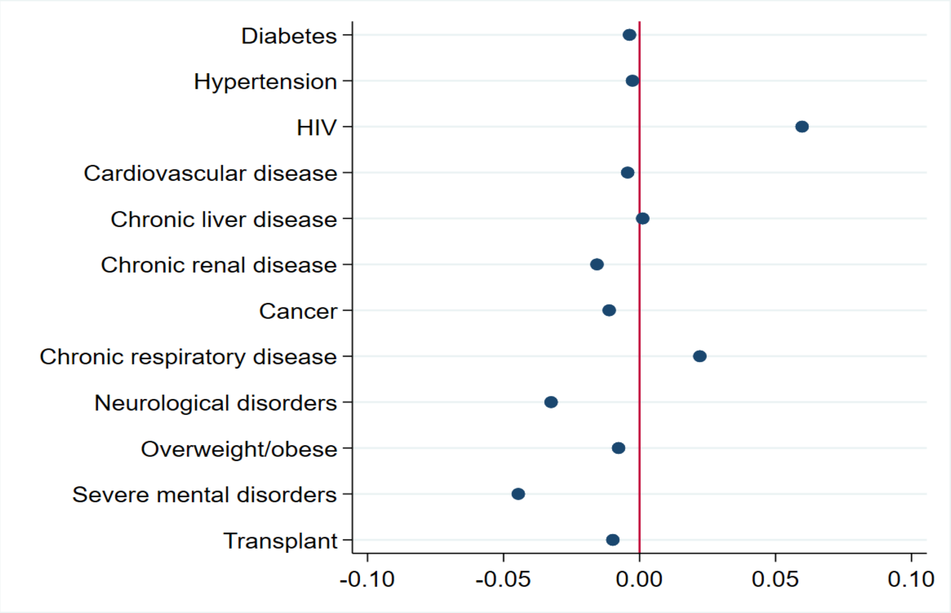

B: Scheme B

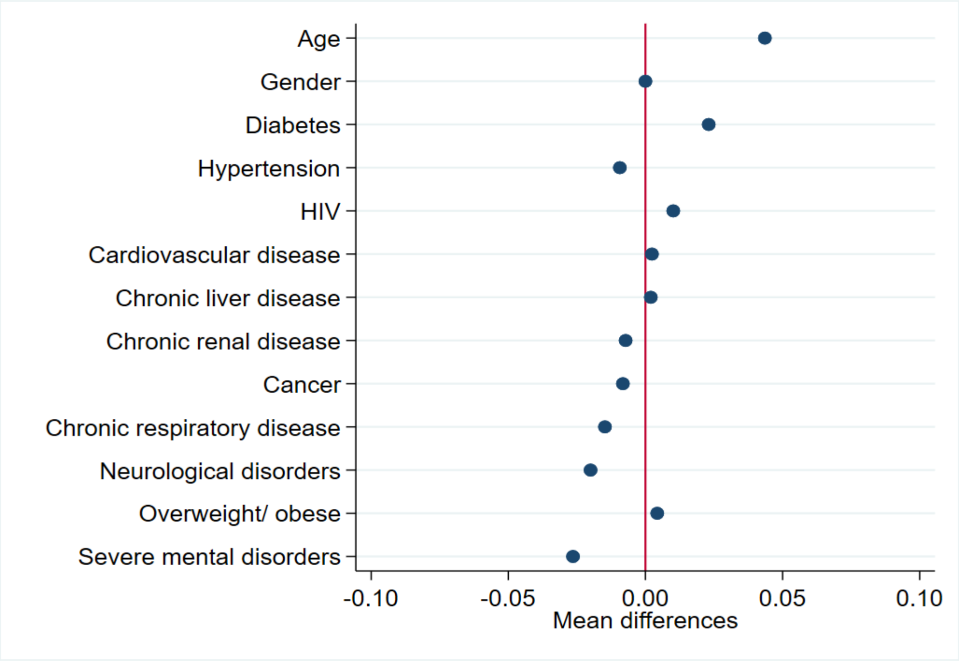

Table S7: COVID-19 event rates and estimated vaccine effectiveness 0-5 days, 6-13 days and 14-27 days after vaccination in scheme participants

Time period	Cohort	COVID-19 hospital admissions			COVID-19 hospital admission requiring critical or intensive care			COVID-19 related death		
		Vaccinated	Unvaccinated	VE (95% CI) %	Vaccinated	Unvaccinated	VE (95% CI) %	Vaccinated	Unvaccinated	VE (95% CI) %
		Events/ P-Y	Events/ P-Y		Events/ P-Y	Events/ P-Y		Events/ P-Y	Events/ P-Y	
0-5 days	Scheme A + B	2/2944	14/2943	84 (50,100)	0/2944	4/2943	-	0/2944	3/2943	-
	Scheme A	1/1352	5/1350	-	0/1352	1/1350	-	0/1352	1/1350	
	Scheme B	1/1592	9/1593	86 (50,99)	0/1592	3/1593		0/1592	2/1593	
6-13 days	Scheme A + B	28/4686	25/4680	-15 (-105,39)	11/4686	7/4680	-97 (-532,50)	0/4686	4/4680	-
	Scheme A	15/2415	12/2137	-34 (-177,42)	2/2145	3/2137	-	0/2145	2/2137	-
	Scheme B	13/2541	13/2543	-11 (-134,56)	9/2541	4/2543	-181 (-1000,37)	0/2541	2/2543	
14-27 days	Scheme A + B	39/7805	58/7785	32 (0,57)	17/7806	18/7787	-5 (-100,48)	5/7807	7/7787	21 (-124,82)
	Scheme A	21/3707	31/3683	30 (-12,61)	7/3708	8/3684	-12 (-186,67)	3/3708	3/3684	-
	Scheme B	18/4098	27/4102	32 (-20,67)	10/4098	10/4103	-17 (-186,61)	2/4099	4/4103	

P-Y: person years; VE: vaccine effectiveness

No estimates were calculated if the minimum number of criteria did not occure during the follow-up period (10 events for schemes; 3 for Western Capre provincial data)

The anomaly around 0-5 days reflects a healthy vaccinee effect where individuals were symptomatically screened and disallowed from vaccination if suspected of having COVID-19. They may also have presented for vaccination if they felt unwell or had been exposed .

Figure S4a: Cumulative incidence of different COVID-19 outcomes in vaccinated and unvaccinated individuals: Scheme A

A. COVID-19 hospital admission

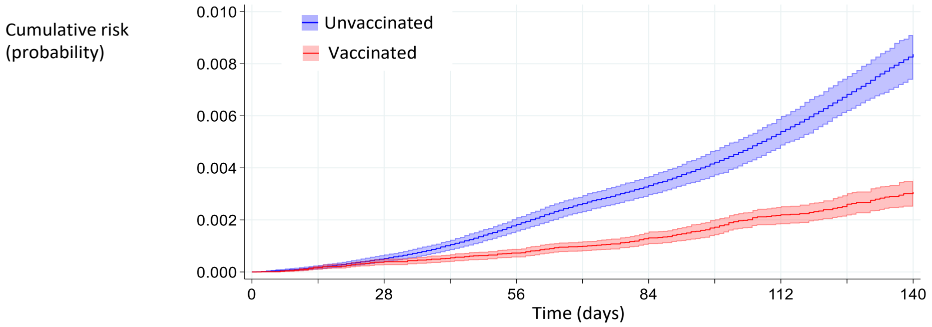

**Number at risk**						
Unvaccinated	99 084	95 334	94 033	56 156	46 444	15 622
Vaccinated	99 084	96 016	95 185	57 493	47 870	16 194
**Cumulative events**						
Unvaccinated	0	50	175	287	398	486
Vaccinated	0	38	70	114	163	188

B. COVID-19 hospital admission requiring critical or intensive care

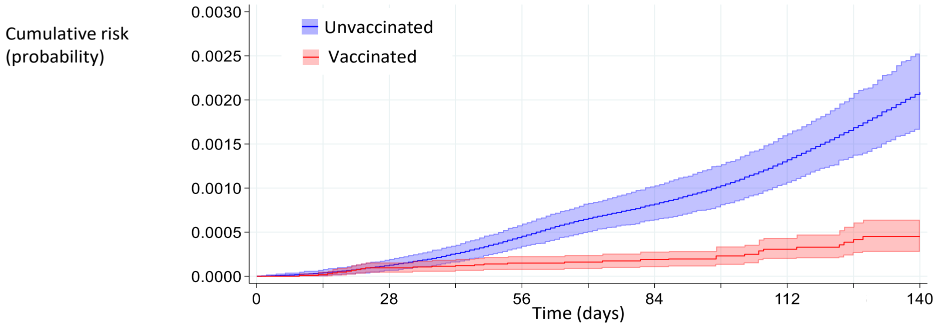

**Number at risk**						
Unvaccinated	99 084	95 370	94 156	56 221	46 558	15 688
Vaccinated	99 084	96 043	95 240	57 533	47 937	16 222
**Cumulative events**						
Unvaccinated	0	12	43	71	98	120
Vaccinated	0	9	14	17	24	29

C. COVID-19 related death

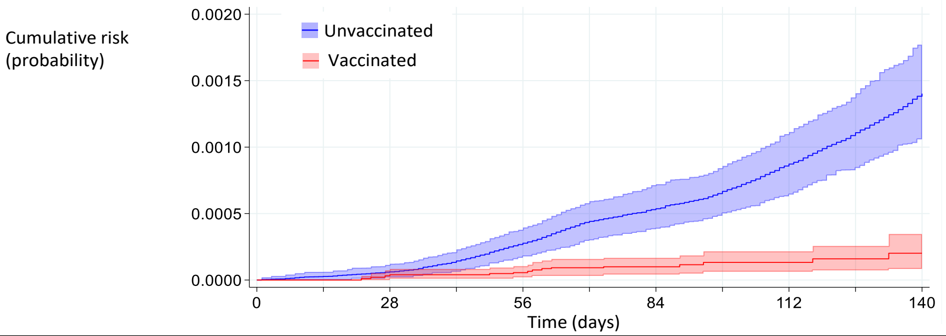

**Number at risk**						
Unvaccinated	99 084	95 378	94 181	56 233	46 577	15 699
Vaccinated	99 084	96 049	95 250	57 535	47 943	16 225
**Cumulative events**						
Unvaccinated	0	6	26	47	65	80
Vaccinated	0	4	5	9	1	13

Figure S4b: Cumulative incidence of different COVID-19 outcomes in vaccinated and unvaccinated individuals: Scheme B

A. COVID-19 hospital admission

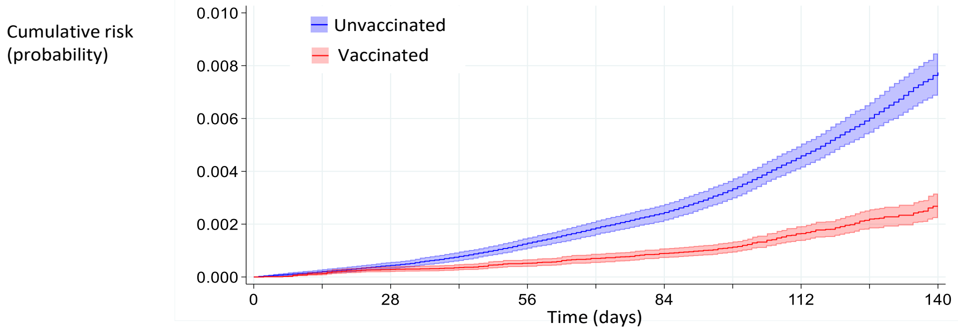

**Number at risk**						
Unvaccinated	116 729	114 586	107 007	81 180	54 190	12 625
Vaccinated	116 729	114 415	106 842	80 963	54 162	12 661
**Cumulative events**						
Unvaccinated	0	51	145	254	403	496
Vaccinated	0	33	59	94	145	176

B. COVID-19 hospital admission requiring critical or intensive care

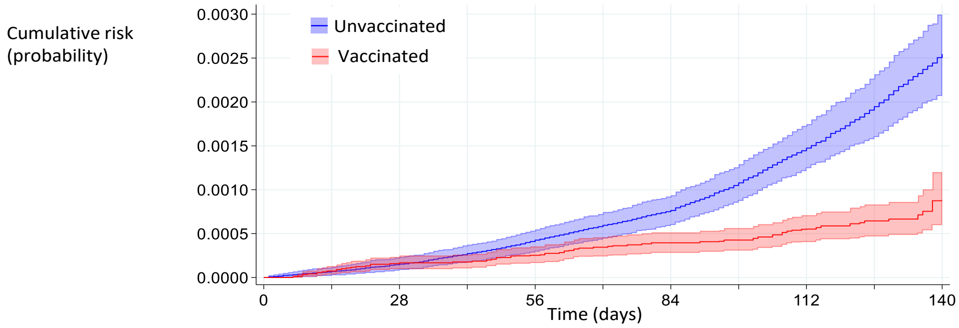

**Number at risk**						
Unvaccinated	116 729	114 617	107 096	81 277	54 330	12 678
Vaccinated	116 729	114 429	106 867	80 989	54 205	12 677
**Cumulative events**						
Unvaccinated	0	18	48	80	130	161
Vaccinated	0	19	29	43	53	61

C. COVID-19 related death

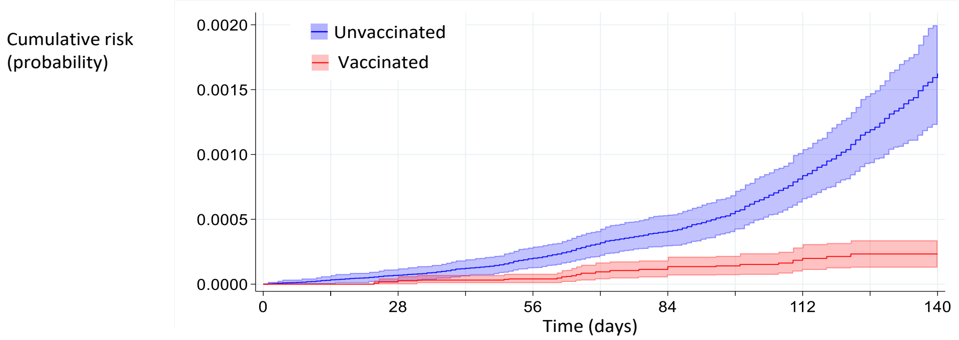

**Number at risk**						
Unvaccinated	116 729	114 633	107 133	81 313	54 382	12 698
Vaccinated	116 729	114 446	106 890	81 015	54 226	12 686
**Cumulative events**						
Unvaccinated	0	8	23	42	72	94
Vaccinated	0	3	5	14	17	19

Figure S4c: Cumulative incidence of COVID-19 related hospitalisation in vaccinated and unvaccinated individuals: Western Cape cohort

A. COVID-19 hospital admission

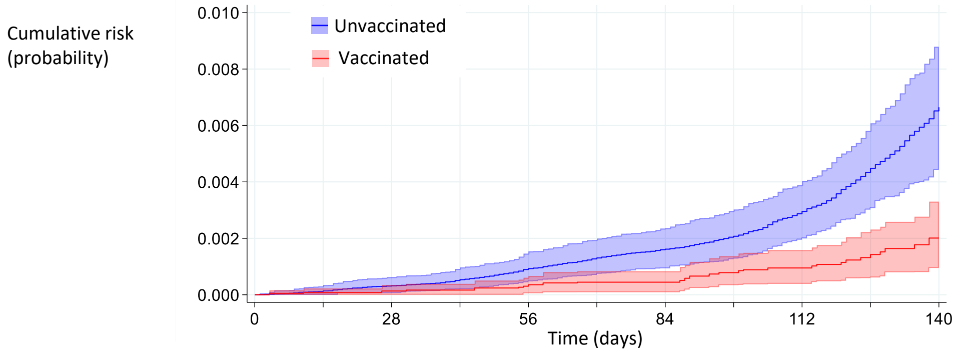

**Number at risk**						
Unvaccinated	19 951	13 236	11 159	9 042	7 049	1 684
Vaccinated	19 951	13 239	11 167	9 054	7 063	1 689
**Cumulative events**						
Unvaccinated	0	5	12	19	30	43
Vaccinated	0	2	4	6	10	14

Table S8: Number of individuals censored for Figure 2 (Cumulative incidence of primary COVID-19 outcomes in vaccinated and unvaccinated insured individuals (schemes A and B) by time since vaccination or matching (A) COVID-19-related hospital admissions, (B) COVID-19-related hospital admissions requiring critical or intensive care, and (C) COVID-19-related deaths.

	Time interval (days)				
	0-27	28-55	56-83	84-111	112-139
A					
Unvaccinated	5792	8561	63162	35901	71405
Vaccinated	5311	8276	63363	36116	72813
B					
Unvaccinated	5797	8643	63603	36384	72241
Vaccinated	5313	8322	63526	36301	73155
C					
Unvaccinated	5789	8647	63680	36451	72388
Vaccinated	5312	8346	63566	36352	73227

Table S9: Number of individuals censored for Figure S4 ((A) COVID-19-related hospital admissions, (B) COVID-19-related hospital admissions requiring critical or intensive care, and (C) COVID-19-related deaths.

Scheme A

	Time interval (days)				
	0-27	28-55	56-83	84-111	112-139
A					
Unvaccinated	3700	1126	37590	9314	30336
Vaccinated	3030	761	37579	9460	31488
B					
Unvaccinated	3702	1171	37864	9565	30750
Vaccinated	3032	789	37690	9572	31686
C					
Unvaccinated	3702	1171	37864	9565	30750
Vaccinated	3032	789	37690	9561	31686

Scheme B

	Time interval (days)				
	0-27	28-55	56-83	84-111	112-139
A					
Unvaccinated	2092	7434	25573	26587	41074
Vaccinated	2281	7514	25785	26656	41435
B					
Unvaccinated	2094	7473	25739	26817	41491
Vaccinated	2281	7533	25835	26731	41467
C					
Unvaccinated	2088	7477	25778	26859	41590
Vaccinated	2280	7551	25861	26772	41521

Western Cape Cohort

	Time interval (days)				
	0-27	28-55	56-83	84-111	112-139
A					
Unvaccinated	6710	2065	2098	1963	5322
Vaccinated	6711	2068	2107	1981	5360
